# Machine Learning Based Prediction of Imminent ICP Insults During Neurocritical Care of Traumatic Brain Injury

**DOI:** 10.1007/s12028-024-02119-7

**Published:** 2024-09-25

**Authors:** Peter Galos, Ludvig Hult, Dave Zachariah, Anders Lewén, Anders Hånell, Timothy Howells, Thomas B. Schön, Per Enblad

**Affiliations:** 1 Division of Neurosurgery, Department of Medical Sciences, Uppsala University, Uppsala, Sweden; 2 Division of Systems and Control, Department of Information Technology, Uppsala University, Uppsala, Sweden

**Keywords:** TBI, AI, Machine learning, Intracranial hypertension, Critical care

## Abstract

**Background:**

In neurointensive care, increased intracranial pressure (ICP) is a feared secondary brain insult in traumatic brain injury (TBI). A system that predicts ICP insults before they emerge may facilitate early optimization of the physiology, which may in turn lead to stopping the predicted ICP insult from occurring. The aim of this study was to evaluate the performance of different artificial intelligence models in predicting the risk of ICP insults.

**Methods:**

The models were trained to predict risk of ICP insults starting within 30 min, using the Uppsala high frequency TBI dataset. A restricted dataset consisting of only monitoring data were used, and an unrestricted dataset using monitoring data as well as clinical data, demographic data, and radiological evaluations was used. Four different model classes were compared: Gaussian process regression, logistic regression, random forest classifier, and Extreme Gradient Boosted decision trees (XGBoost).

**Results:**

Six hundred and two patients with TBI were included (total monitoring 138,411 h). On the task of predicting upcoming ICP insults, the Gaussian process regression model performed similarly on the Uppsala high frequency TBI dataset (sensitivity 93.2%, specificity 93.9%, area under the receiver operating characteristic curve [AUROC] 98.3%), as in earlier smaller studies. Using a more flexible model (XGBoost) resulted in a comparable performance (sensitivity 93.8%, specificity 94.6%, AUROC 98.7%). Adding more clinical variables and features further improved the performance of the models slightly (XGBoost: sensitivity 94.1%, specificity of 94.6%, AUROC 98.8%).

**Conclusions:**

Artificial intelligence models have potential to become valuable tools for predicting ICP insults in advance during neurointensive care. The fact that common off-the-shelf models, such as XGBoost, performed well in predicting ICP insults opens new possibilities that can lead to faster advances in the field and earlier clinical implementations.

**Supplementary Information:**

The online version contains supplementary material available at 10.1007/s12028-024-02119-7.

## Introduction

Traumatic brain injury (TBI) affects 50–60 million people globally each year, with an annual cost of around US $400 billion. In Europe, every year, about 82,000 people die and 2 million are admitted to a hospital [1]. The primary brain injury caused by the trauma renders the brain vulnerable for secondary insults, which in turn may cause secondary brain injury. Treatment of TBI is therefore focused on avoiding secondary insults, for example, raised intracranial pressure (ICP), hypotension or fever. Using this strategy, neurointensive care (NIC) has substantially improved outcome [2–4]. One of the most severe secondary insults is increased ICP. Monitoring of ICP is therefore crucial in NIC, in which intraparenchymal sensors or ventricular drain catheters are used for continuous measurement. The goal is usually an ICP below 20 mm Hg. When ICP increases over this level, an alarm is triggered.

An ability to be able to predict and give warning about upcoming episodes of harmful ICP (i.e., potential ICP insults) would be of great importance. Patients with increased risk of an imminent ICP insult could be observed more closely. Physicians on call can be made aware, and diagnostic procedures could, if needed, be initiated at an early stage. A system that successfully predicts ICP insults before they emerge may facilitate early optimization of the physiology, which may in turn lead to that the ICP insult will never occur, and thus secondary brain injury never develops.

Artificial intelligence (AI) may have an important role in health and medicine. Research has shown its potential usefulness in, for example, radiology, pathology, gastroenterology, and ophthalmology. Using rich data sources, machine learning (ML) methods have been used to predict outcome from medical signal data [5].

There are expectations that AI may become a valuable tool for predicting ICP insults of patients with TBI in advance, and several attempts have been published, which are well-summarized in two recent review articles [6, 7]. The results appear promising from a clinical perspective. However, the studies are heterogenous, with differences in number of patients, definitions of ICP insults, sampling frequencies, and performance measures. Before the implementation of a prediction model in the clinical setting, there is a need for more studies evaluating AI models in larger TBI cohorts with substantial amounts of collected monitoring data. The TBI registry in Uppsala [8] combined with prospectively collected high-resolution monitoring data from more than 600 patients provides an opportunity for a study of this kind.

The aim of this study was therefore to evaluate the performance of different AI models on the Uppsala dataset and compare them to promising models from similar previously published larger studies [9–15].

## Methods

### Patients and Management Protocol

The neurointensive care unit at Uppsala University Hospital provides specialized care for the Uppsala region and neighboring regions with a combined population of 2 million (longest distance admitting local hospital 300 km). The patients with TBI in this study had to fulfill three inclusion criteria: (1) Age 16 years or older, (2) Treated in Uppsala university hospital NIC unit between 2008 and 2019 and (3) At least 10 h of ICP monitoring time.

The NIC unit follows standardized protocols emphasizing that prevention and intensive treatment of secondary insults are of utmost importance to avoid secondary brain injury [4]. Briefly, all patients not responding to commands (Glasgow coma motor score 1–5) are mechanically ventilated and have ICP monitoring. Mechanically ventilated patients receive propofol for sedation and morphine as analgesia. For ICP monitoring, a ventricular drainage catheter (HanniSet, Xtrans, Smith Medical GmbH, Glasbrunn, Germany or VentrEX, Neuromedex, Hamburg, Germany) is used as first choice or alternatively an intraparenchymal sensor (Codman ICP Micro-Sensor, Codman and Shurtleff, Raynham, MA) if the ventricular system is compressed. ICP should be treated in an escalatory manner using for example evacuation of mass lesions, increased sedation, cerebrospinal fluid (CSF) drainage, hyperventilation, barbiturates and/or decompressive craniectomy. The goal is to keep ICP below 20 mm Hg and cerebral perfusion pressure above 60 mm Hg (50 mm Hg during barbiturate coma).

### Dataset

High-frequency (100–200 Hz) physiological patient monitoring data, such as invasive arterial blood pressure (ABP) and ICP, is routinely collected from the bedside monitor by the ODIN system, a data monitoring and collection system developed in Edinburgh and Uppsala by Tim Howells et al. [16]. Additionally, for all patients with TBI treated in the NIC unit, clinical and demographic data, radiological evaluations and outcome scores, are stored in the Uppsala Clinical Research Center (UCR) TBI database [8].

For this study, high frequency monitoring data from the ODIN database were merged with clinical data from the UCR TBI database. No artifact cleaning or other preprocessing was performed. The resulting dataset consisted of 1125 eligible patients, out of whom 612 fulfilled the inclusion criteria. During initial analysis, 10 patients were removed, due to missing clinical variables on arrival (3), no registered result from CT-scan on arrival (4), missing ECG data (2) or missing ABP data (1). In the end, 602 patients were included in the study. This dataset is referred to as UHF-TBI dataset, the Uppsala High Frequency TBI dataset.

Out of hospital large-scale high-performance computing resources were used for AI model evaluation: The Swedish National Infrastructure for Computing (SNIC) and The National Academic Infrastructure for Supercomputing in Sweden (NAISS). The data were anonymized according to best practice before transfer to SNIC/NAISS to fulfill legal requirements.

### Predictive Task, Definition of an ICP Insult and Physiological Features

Earlier research in predicting ICP insults have shown good results using a window of a few hours to predict upcoming insults [9]. Increasing the size of the historical window beyond 4–6 h results only in marginal improvements [17]. Therefore, as in previous studies [9, 12], a 4 h historical window was used, followed by a 30-min prediction window, comprising nonoverlapping 4.5-h time sections. The task of the trained models was to predict an ICP insult starting within 30 min after the historical window.

**Fig. 1:**
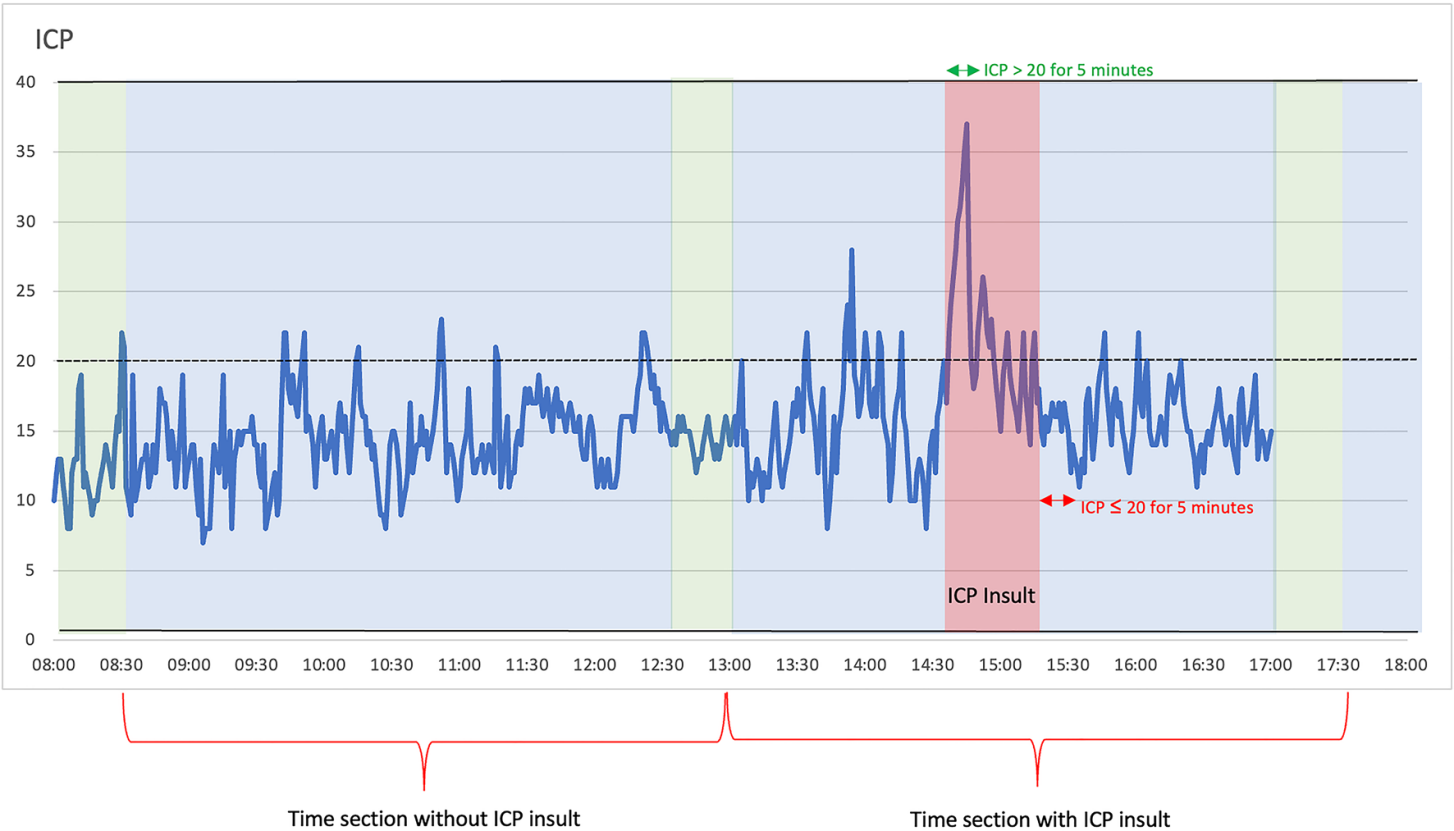
Two consecutive time sections (blue is historical data, green is prediction), one with and one without an ICP insult according to the definition applied. ICP insult Definition: ICP above 20 mmHg for 5 min marks the beginning of an ICP insult. The timespan of the ICP insult stretches from the start of such episode until the beginning of a period when ICP is below or equal to 20 mmHg for another 5 min (Color figure online)

Different definitions of ICP insults have been used in earlier studies, such as an ICP of at least 22 mmHg lasting for at least 5 min [18] or 30 mmHg for 10 min [9]. However, using definitions with strict ICP thresholds, potential insults could be missed due to a single subthreshold ICP value in the range. Therefore, in this study an ICP above 20 mm Hg for 5 min marks the beginning of an ICP insult. The timespan of the ICP insult stretches from the start of such episode until the beginning of a period when ICP is below or equal to 20 mmHg for another 5 min (Fig. 1) [19].

There are a number of possible predictors of ICP during TBI intensive care, that we refer to as physiological features. Examples of features are the current ICP value, heart rate and the correlation between ICP and ABP. Identifying and selecting the most important features may improve model performance and make the model more interpretable in clinical terms. In cases of missing data, the value “0” was imputed, and a feature indicating missingness was introduced. The data and features used in this study are presented in Appendix.

### Evaluation of Model Performance

Various evaluation metrics for ML models, emphasizing different aspects of model performance, exists. Clinically relevant metrics must be chosen [20], while acknowledging that a single summary measure cannot fully describe model performance [21, 22]. We have chosen to evaluate model discriminatory power in three ways. (1) Sensitivity (or recall), i.e. the ratio of ICP-alarms to total actual ICP insults in the data and specificity, i.e. the fraction of no ICP alarm when there is no actual ICP insult. (2) The ROC (receiver operating characteristic curve), which describes the performance of a binary classification model, at all classification thresholds. Each model will return a predicted score between 0 and 1. The model assigns the positive class (insult) if the score is above some threshold value, otherwise it assigns the negative class (no insult). The default classification threshold is 0.5 to separate insult from no insult. By varying the threshold, a curve can be plotted with the false positive rate on the *X* axis and the true positive rate on the *Y* axis. The area under ROC (AUROC) can therefore be seen as an aggregate measure of the performance across all possible thresholds and is a frequently used indicator of ML model performance. (3) Balanced accuracy, the arithmetic mean of the sensitivity and the specificity. All three performance metrics are independent of the underlying insult rate.

Two complementary qualities for clinical prediction models are Calibration and Net Benefit [20]. Since the primary evaluation metrics in this study are balanced metrics—independent of the underlying insult rates—we expect models optimized for balanced metrics to show some miscalibration. Brier score will be computed and presented as well as calibration plots. Net Benefit curves aims at quantifying the benefit of the model, by parametrizing the trade off between True Positive prediction and False Positive prediction using a probability at which a physician or patient is indifferent to stating a diagnosis versus not [23]. The methodology seem to be designed for calibrated models, and may have limited applicability to our models, but will be presented for completeness. Clinical discussions set the range of relevant thresholds *p*_*t*_ to 0–25%.

### AI Models Used in Predictive Task

*Four different model classes were used* Gaussian process regression, Logistic Regression, Random Forest classifier and Extreme Gradient Boosted Decision Trees. All software packages were obtained from PyPI [24] and the code was implemented with Python 3.8 [25].

Two different subclasses of models, unrestricted (-U) and restricted (-R), were used in the study. The *unrestricted models* include all UHF-TBI dataset variables, i.e. both arrival and monitoring data. Several physiological features were calculated to extract high frequency information, and was then downsampled to minute-means: heart rate, respiratory rate (calculated from ECG), heart rate variability (defined as short term 5-min standard deviation of R-R intervals), and mean/systolic/diastolic arterial and intercranial pressures. Length of monitoring (time since start of monitoring), was also included for the unrestricted models. The *restricted models* include the same features as the Gaussian process (GP) model developed by Güiza et al. [9]. Details about the features are presented in Appendix.

The models were evaluated by *year wise cross-validation* [26]. With a total of 12 years of data, each year in the data was assigned as test data, and the other 11 years as training data. Doing this for all 12 years, 12 years-folds were defined. For each fold, the performance metrics were calculated and presented as an average with standard deviation.

The default classification threshold was used, as well as a tuned and a 90 threshold, respectively. The tuned threshold was defined as the threshold creating the largest balanced accuracy in the training set. The 90 threshold was defined as the threshold that resulted in a 90% sensitivity in the training set. We found that a tuned classification threshold gave rise to higher standard deviation of the balanced accuracy/sensitivity across year-folds; which may indicate overfitting (Supplement Table 1). Therefore the default threshold (0.5) was used.

**Table 1:** Demographics and arrival variables for the patients included in the study

Demographic categories	*n*	Percentage
Total	602	100
Male	469	78
Age (*y*)		
16 to 20	62	10
21 to 25	48	8
26 to 30	34	6
31 to 35	24	4
36 to 40	34	6
41 to 45	33	5
46 to 50	46	8
51 to 55	56	9
56 to 60	49	8
61 to 65	67	11
66 to 70	64	11
71 to 75	49	8
75 +	36	6
Arrival variables		
CT findings		
Acute SDH	175	29
Contusions	155	26
Mixed	119	20
DAI	48	8
Traumatic SAH	40	7
EDH	37	6
Other	16	3
Impression fracture	12	2
Pupil right		
Normal	390	65
Sluggish	140	23
Fixed	72	12
Pupil left		
Normal	388	64
Sluggish	137	23
Fixed	77	13
GCS		
* Eye response*		
1, no eye opening	349	58
2, eye opening to verbal command	80	13
3, eye opening to pain	64	11
4, eyes open spontaneously	60	10
Not applicable	49	8
Verbal response		
1, no verbal response	160	27
2, incomprehensible sounds	12	2
3, inappropriate words	2	0
4, confused	29	5
5, oriented	23	4
Not applicable	376	62
Motor response		
1, no motor response	26	4
2, extension to pain	23	4
3, flexion to pain	32	5
4, withdrawal from pain	113	19
5, localizing pain	242	40
6, obeys commands	166	28
RLS-85		
1	8	1
2	57	9
3a	96	16
3b	191	32
4	89	15
5	86	14
6	25	4
7	24	4
8	26	4
Anticoagulation	97	16

CT, computed tomography, DAI, xxx, EDH, xxx GCS, Glasgow Coma Scale, SAH, xxxx, SDH, xxx

### Gaussian Process Regression

The restricted Gaussian process regression model (GP-R) was built on the restricted features, using the same variables as Güiza et al. [9, 12]. Data were first standardized. After that, the correlation-based feature-selection algorithm [27] was applied, using the Pearson correlation coefficient and a best-first-search. Then, the training data were split into proper training data (90%) and validation data (10%) stratified by class (insult/no insult). A variational Gaussian process was fit with Pólya-Gamma data augmentation and inducing points [27]. Five thousand inducing points was chosen to provide a tradeoff between computational burden and model performance, also being larger than the number of data points in the work by Güiza et al. [9]. To promote class-balanced performance, the inducing points were initialized at all the *p* positive class label data points (n.b. *p* < 2,500), and at 5000-*p* randomly sampled negative class label data points. The variational parameters were minimized by natural gradient descent, and all other parameters were optimized with Adam [28], to minimize the variational ELBO. The data were sampled with inverse class frequency probability, ensuring the variational ELBO was class balanced. The libraries GPyTorch 1.11 and PyTorch 1.12 were used. The correlation-based feature-selection algorithm was developed locally according to Hall [29]. Forty-five epochs of training were done for each year-fold. Convergence and absence of overfitting was verified on the validation data set.

### Logistic Regression

The logistic regression model (LR-U) was fit with L1-penalty on the unrestricted feature set. The logistic loss was class balanced per training fold. The penalization was selected by fivefold cross-validation (CV), grouped by patient to avoid data leakage due to patients being present in train data and validation data. While year-grouped CV seems more accurate for the generalization task of prediction to an unseen year, this would lead to too large groups for fivefold CV, and that patient-grouped CV constituted a suitable trade off. For the optimal regularization, maximizing the balanced accuracy, the model was refit on the full data set. The logistic regression model was implemented with scikit-learn version 1.2.2 [30].

### Random Forest Classifier

The two random forest models, (RF-R, and RF-U) differ only on whether the feature set was restricted or unrestricted. One thousand decision trees were fitted, otherwise using default parameters for random forest classifiers, from the software library scikit-learn version 1.2.2 [30]. Notably, the trees were split into one sample per leaf. Each tree had access to a random subset of features and a bootstrapped dataset. Splitting decisions were taken to minimize the class-balanced Gini impurity.

### Extreme Gradient Boosting

Analogously to the random forest classifier, the extreme gradient boosting models (XGBoost-R and XGBoost-U) differ only on whether the feature set was restricted or unrestricted. Both use extreme gradient boosted decision trees from the software library XGBoost 1.5.2 [31]. Patient-grouped fivefold CV identical to the one for the Logistic Regression model was employed to find hyperparameters. The optimization objective was the class-balanced negative log likelihood. Hyperparameter search was made over tree height, learning rate, feature subsampling and number of trees in the ensemble. The tree method was histogram-based to allow for graphics processing unit acceleration. Other parameters were kept to their as default values.

### Ethics

The study was approved by the National Ethical Review Authority. Informed consent was obtained from the patient, or next of kin if the patient was unable to give consent. This consent is valid for analysis of collected data generated during neurointensive care for different future research questions. The Declaration of Helsinki and its subsequent revisions were followed.

## Results

### Patient Characteristics and ICP Insults

The results are based on 602 patients with TBI distributed over 12 years, with an average of 50 patients per year (min 37, max 75, SD 10.3). There were 78% men with a mean age of 46 years. The patient characteristics are detailed in Table 1.

The average length of NIC monitoring was 9.6 days. Total patient monitoring time of the study was 138 411 h. During that time, 1925 of the 4.5-h nonoverlapping time sections had an insult within 30 min, which constitutes a prevalence of 6.3% among the 30,758 time sections. The number of time segments and insults per year is presented in Table 2. The prevalence varies between 2.5% to 13.6% over the years.

**Table 2:** Prevalence of insults over study years

Year	Time sections (n)	Time section w insult (n)	Insult rate
A	3071	417	13.6%
B	2353	85	3.6%
C	2465	182	7.4%
D	3852	232	6.0%
E	2168	99	4.6%
F	2797	165	5.9%
G	2511	124	4.9%
H	2131	69	3.2%
I	2058	52	2.5%
J	1994	234	11.7%
K	2896	107	3.7%
L	2462	159	6.5%
Total	30758	1925	6.3%

Years are not necessarily in chronological order

### The Performance of the Different Models

The metrics for the different models are presented in Table 3. Using sensitivity/specificity as performance measure, XCBoost-U performed best with a sensitivity of 94.1% (SD 3.5%) and specificity of 94.6% (SD 2.2%), followed by XCBoost-R (sensitivity 93.8% and specificity 94.6%), GP-R (sensitivity 93.2% and specificity 93.9%) and RF-R (sensitivity 60.4% and specificity 99.3%).

**Table 3:** Results from the different models using different evaluation metrics

Variable/model	GP-R	LR-U	RF-R	RF-U	XGBoost-R	XGBoost-U
AUROC, mean (SD) (%)	98.3 (0.57)	97.1 (0.84)	98.7 (0.49)	98.7 (0.45)	98.7 (0.53)	98.8 (0.45)
Sensitivity, mean (SD) (%)	93.2 (3.32)	99.5 (0.79)	60.4 (7.72)	57.9 (7.75)	93.8 (3.68)	94.1 (3.51)
Specificity, mean (SD) (%)	93.9 (2.22)	56.1 (10.29)	99.3 (0.33)	99.4 (0.28)	94.6 (2.27)	94.6 (2.24)
Brier score, mean (SD) (%)	0.04 (0.01)	0.25 (0.04)	0.02 (0.01)	0.02 (0.01)	0.04 (0.01)	0.04 (0.01)
Accuracy, mean (SD) (%)	94.0 (1.92)	59.0 (8.37)	97.0 (1.32)	96.9 (1.39)	94.6 (1.98)	94.7 (1.97)
Balanced Accuracy, mean (SD) (%)	93.6 (1.18)	77.8 (4.95)	79.9 (3.88)	78.6 (3.88)	94.2 (1.71)	94.3 (1.67)
f1, mean (SD) (%)	63.9 (5.89)	21.7 (6.4)	70.2 (7.55)	68.8 (7.17)	66.8 (5.55)	67.1 (5.99)
PPV, mean (SD) (%)	48.7 (6.37)	12.3 (4.23)	84.4 (9.01)	85.6 (7.75)	52.0 (5.98)	52.3 (6.62)

Five supplementary metrics are presented: PPV, f1, accuracy (class-weighted mean of sensitivity and specificity), balanced accuracy (the arithmetic mean of the sensitivity and the specificity), and Brier score. AUROC, area under the receiver operating characteristic curve, f1, harmonic mean of the PPV and sensitivity, GP, Gaussian process regression, LR, logistic regression, PPV, positive predictive value, RF, random forest classifier, SD, standard deviation, XGBoost, Extreme Gradient Boosted decision trees

**Fig. 2:**
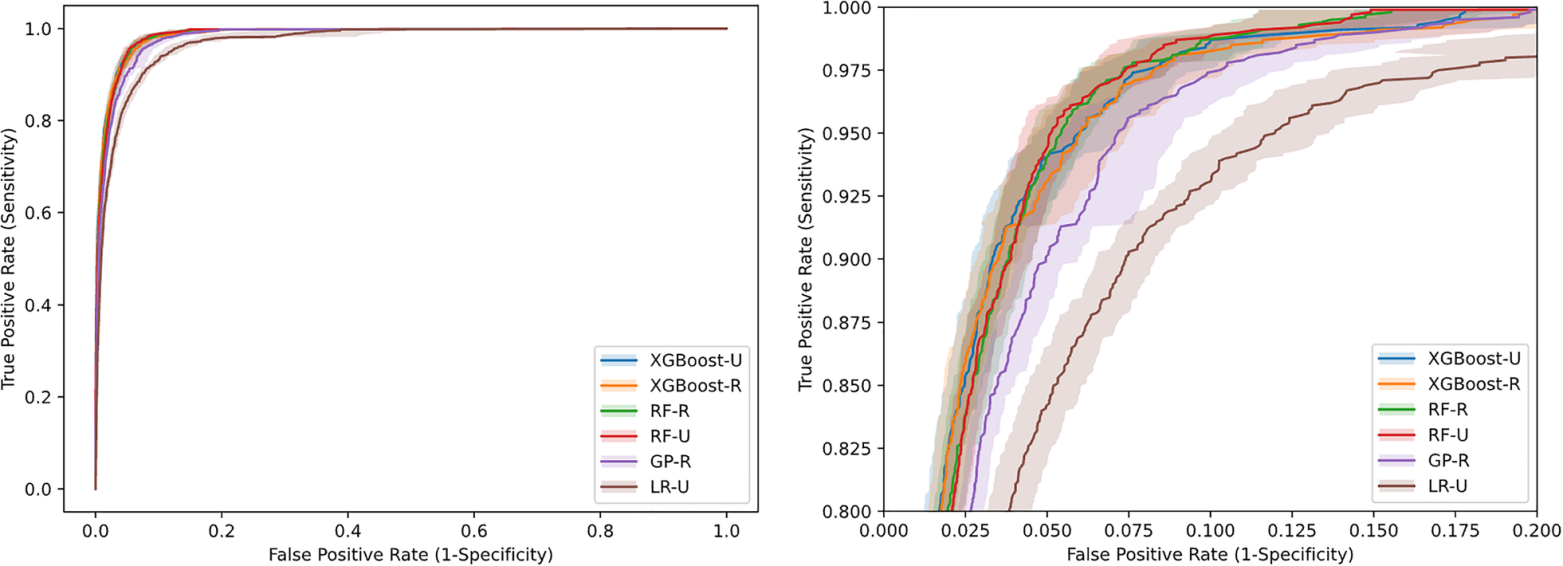
Average ROC curves with 1.96 standard error uncertainty bands, original and zoomed in. The curves describes the mean and the spread in false positive rate given some true positive rate [33]

There was little difference in the AUROC performance of the models (Table 3 and Fig. 2). Top models were XGBoost-U (98.8%, SD 0.5%), XGBoost-R (98.7%, SD 0.5%), RF-R/-U (98.7%, SD 0.5%) and GP-R (98.3%, SD 0.6%).

Random forests RF-R/-U show best Brier score (0.02). All other models, which are optimized for class-balanced log likelihood, show overestimation of probability of insult, based on the calibration plots (Supplement Figs. 1–12). The Net Benefit curves show Net Benefit over the range of plausible thresholds *p*_*t*_, with Random forests generally giving best Net Benefit (Supplement Figs. 13–24).

## Discussion

We have trained predictive models on the UHF-TBI dataset to forecast upcoming ICP insults. The ultimate goal is to obtain a clinically valuable AI tool for insult risk prediction, which should provide an opportunity to significantly reduce the risk and prevent that the ICP insult occur. In the effort of developing a clinical AI tool for prediction of an upcoming ICP insult there are some methodological issues that needs to be discussed overall and in perspective of earlier TBI studies, summarized in Table 4.

**Table 4:** Comparison of studies using AI for prediction of ICP insults in patients with TBI

Study	Reference	Comment	n	Dataset	Sampling frequency	ICP insult definition	Model	AUROC	Sensitivity	Specificity	Accuracy	f1	PPV
Güiza 2013	9	Original GP model. 30 min in advance based on 4 h of past ICP and ABP	239	Brain-IT	< = 1/60 Hz	30 mmHg ≥ 10 min	GP	87.0%	82%	75%	77%		
Myers 2016	10	15–360 min prediction of ICP crisis based on 30 min past data	817	Ben Taub Hospital, USA	1/36 Hz	≥ 20 mmHg ≥ 15 min	AR-OR	86.0%	-	-	-		
Güiza 2017	11	External validation of Güiza 2013 (in this table adult cohort only)	121	5 centers		> 30 mmHg ≥ 10 min	GP	90.0%	70%	90%	86%		
Carra 2021	12	External validation of Güiza 2013	257	CENTER TBI	> 100 Hz, 1/60 Hz	30 mmHg ≥ 10 min	GP	93.0%	83%	91%	88%		
Wijayatung 2022	13	10-min intervals during 60 min, based on preceding hour of ICP	29	Umeå university hospital, Sweden	125 Hz	ICP ≥ 20 mmHg	Naïve Bayes	-	87%	95%	95%		
Petrov 2023	14	10–20-min intervals based on preceding 1–2 h of data	36	Upenn Health System, USA		ICP > 22 mmHg for ≥ 75% in a 5-min interval	RF	88.0%	46%	-	86%	57%	76%
Carra 2023	15	Early prediction of doses of harmful intracranial pressure (development/validation)	554	BrainIT + CENTER TBI		ICP > 22 mmHg	RF, multiple GP	94.0%	78%	94%	89%		
This study			602	UHF-TBI	200 Hz, 100 Hz, 1/60 Hz	Start: > 20 mmHg > 5 min, stop: ≤ 20 mmHg > 5 min	Dummy	50.0%	0.0%	100.0%	93.9%	0.0%	-
							GP-R	98.3%	93.2%	93.9%	94.0%	63.9%	48.7%
							LR-U	97.1%	99.5%	56.1%	59.0%	21.7%	12.3%
							RF-R	98.7%	60.4%	99.3%	97.0%	70.2%	84.4%
							RF-U	98.7%	57.9%	99.4%	96.9%	68.8%	85.6%
							XGBOOST-R	98.7%	93.8%	94.6%	94.6%	66.8%	52.0%
							XGBOOST-U	98.8%	94.1%	94.6%	94.7%	67.1%	52.3%

For details, see Appendix. AR-OR, autoregressive ordinal-regression, GP, Gaussian process regression, LR, logistic regression, R, restricted dataset (physiological data only), RF, random forest, TBI, traumatic brain injury, U, unrestricted dataset (physiological data + other data), XGBOOST, Extreme Gradient Boosted decision trees

It is crucial to define a clinically relevant ICP insult which also is manageable for the model. The choice of our ICP threshold corresponded to the relatively established NIC target to keep the ICP below or equal to 20 mmHg and we found a higher ICP level less clinically relevant. When ICP is above 20 mmHg for longer periods than around 5 min, one can anticipate that the risk of developing secondary brain injury becomes significant. Applying a definition of ICP more than 20 mm Hg for 5 min yielded quite few insults in the UHF-TBI dataset (ICP > 20 mmHg 6.2% of the monitoring time). Too few insults in the training data would make the ML more difficult. Another thing to consider is how the insult definition should reflect situations when ICP is above 20 mmHg for significant periods but with short dips under or equal to 20 mmHg, which could be ascribed to be multiple insults while it more has the character of a longer single insult. That was another the reason why we did not use the 5 min duration criteria and instead defined an end of the ICP insult as ICP below or equal to 20 mmHg for 5 min (Fig. 1).

In the first model (GP-R), we tried to replicate the Gaussian process model by Güiza et al. [9] as far as possible, as a reference for comparison. The Güiza model was chosen since it was trained and validated on a relatively large datasets of only patients with TBI and externally validated [11, 12]. The correlation-based feature-selection was implemented and worked well in our code. Pearson correlation and forwards-selection of variables were performed, although not specified in the original paper. Since the exact GP is computationally very expensive we opted for the computationally faster variational GP. Comparison of different classification thresholds showed that threshold tuning on the training data did not yield the performance boost that was intended. The ambition was to increase the balanced accuracy on test data, but it instead decreased. Thus, the GP-R model applied in our study is generally similar to the Güiza model [9] to a large extent, although differences exists. The results showed that our GP-R model based on the Güiza model [9] performed well (AUROC = 98.3%, Sensitivity = 93.2%, specificity = 93.9%) on the UHF-TBI dataset, despite applied on a different dataset, with different insult definitions and some model differences.

Switching to the unrestricted feature set in the flexible models increased performance slightly. Hence, the prediction based on restricted monitoring data set may be improved by adding e.g. ECG, demographics and arrival data (Appendix). With the aim of predicting an upcoming ICP insult, categorical data need to be simple to add in the clinical prediction tool upon patient arrival. It is also important to consider that using more features will increase the computational burden. All models considered in this work are still lightweight enough to be deployed to a desktop computer, even if the models were originally trained on a high-performance computing facility. Furthermore, there is a need for proper regularization of models to avoid overfitting. We have controlled this via the year-fold CV. For the unrestricted RF model some deterioration was found when increasing the number of features, and we interpret this to be caused by insufficient regularization.

Model evaluation was primarily done on the basis of AUROC, sensitivity and specificity, since these are core classifier performance metrics for binary predictive models invariant to changes in prevalence. The calibration of the models were moderate to poor as expected—the optimization targets (balanced log likelihood and gini impurity) aims at improving the balanced accuracy rather than the calibration. All models except LR showed positive Net Benefit for *p*_*t*_ over the whole range 0–25%, but with substantial variability. One source of variation is the variation in prevalence—Net Benefit curves vary with the prevalence [32]. Considering the poor calibration the heuristic of chosing the decision threshold of *p*_*t*_ mandated by the Net Benefit literature may be inappropriate, and models may show greater Net Benefit if using an optimized decision threshold instead.

We have in this study analyzed model performance on historical data. Model performance in deployment depends on similarities between historical and future data. Relatively small variance per year indicates that it should extrapolate well to future data. Due to anonymized year codes, we cannot say if there is temporal trend in performance. The ability to generalize to other hospitals is not possible to assess without an external dataset, and therefore not addressed in this paper. The fact that a common off-the-shelf model such as XGBoost performed well in predicting ICP insults opens for new possibilities; the rich ecosystem of services, such as Automated ML (AutoML) for training standard models, can lead to faster advances in the field and faster clinical implementations. One possibility is also to use more advanced models, such as convolutional neural networks (deep learning) and transfer learning models, in order to improve prediction performance.

## Limitations

Despite promising model performances, several aspects must be studied further before clinical implementation. The models do not have access to any information about treatment decisions, so any prediction made by the models may be unreliable if the treatment protocol is changed. Collecting treatment data with time stamps [19] is one step forward to enable decision supporting prediction models. Using ML explainability tools such as SHAP or LIME can also be applied to shed light on the mechanisms for prediction. For generalized use of any model in other hospitals, the performance of the model also needs to be validated on an external TBI dataset. Another limitation is that we have not studied the model’s performance with respect to patient subgroups. There is a gender imbalance in the data, which could cause unfair prediction performance. Other important variables such as ethnicity are not recorded, and it is a limitation of our database that we cannot study AI fairness aspects fully.

## Conclusions

The GP model has been successfully applied to the task of predicting upcoming ICP insults (sensitivity and specificity around 93%), in accordance with earlier studies [9, 11, 12]. Using a more flexible model (XGBoost) on the UHF-TBI dataset provided comparable performance related to the variational GP model. In addition, this model is generally more available and easily applied. Adding more clinical variables and physiological features improved the performance of the models somewhat (sensitivity and specificity above 94%). A clinical tool that can predict an upcoming ICP insult with > 94% sensitivity and specificity would be of great use in the NIC setting.

## Electronic supplementary material

Below is the link to the electronic supplementary material.

Supplementary file1 (DOCX 18 KB)

Supplementary file2 (PDF 21 KB)

Supplementary file3 (PDF 20 KB)

Supplementary file4 (PDF 21 KB)

Supplementary file5 (PDF 21 KB)

Supplementary file6 (PDF 21 KB)

Supplementary file7 (PDF 21 KB)

Supplementary file8 (PDF 21 KB)

Supplementary file9 (PDF 21 KB)

Supplementary file10 (PDF 20 KB)

Supplementary file11 (PDF 22 KB)

Supplementary file12 (PDF 20 KB)

Supplementary file13 (PDF 21 KB)

Supplementary file14 (PDF 20 KB)

Supplementary file15 (PDF 20 KB)

Supplementary file16 (PDF 21 KB)

Supplementary file17 (PDF 21 KB)

Supplementary file18 (PDF 20 KB)

Supplementary file19 (PDF 21 KB)

Supplementary file20 (PDF 20 KB)

Supplementary file21 (PDF 20 KB)

Supplementary file22 (PDF 19 KB)

Supplementary file23 (PDF 20 KB)

Supplementary file24 (PDF 20 KB)

Supplementary file25 (PDF 21 KB)

Supplementary file25 (TIFF 3309 KB)

Supplementary file27 (DOCX 28 KB)

Supplementary file28 (DOCX 455 KB)

## Appendix

Features and Data.
