# Supplementary material for: Machine Learning Based Prediction of Imminent ICP Insults During Neurocritical Care of Traumatic Brain Injury
Source: Neurocrit Care. 2024 Sep 25;42(2):387–97. doi: 10.1007/s12028-024-02119-7 (PMC11950052; doi:10.1007/s12028-024-02119-7)
Supplement: Supplementary file 1 — Supplementary file1 (DOCX 18 KB) [file 12028_2024_2119_MOESM1_ESM.docx]

Supplement Figs. 1–12 **Calibration plots for Hold-out year 1­–12.**

Supplement Figs. 13–24 **Net Benefit curves for Hold-out year 1–12.**

Supplement Table 1 Comparison of different thresholds. Default threshold was 0.5. Tuned threshold was defined as the threshold creating the largest sum of sensitivity and specificity in the training set. The 90 threshold was defined as the threshold that resulted in a 90% sensitivity in the training set.
