# Supplementary figures and images for: Machine Learning Based Prediction of Imminent ICP Insults During Neurocritical Care of Traumatic Brain Injury
Source: Neurocrit Care. 2024 Sep 25;42(2):387–97. doi: 10.1007/s12028-024-02119-7 (PMC11950052; doi:10.1007/s12028-024-02119-7)

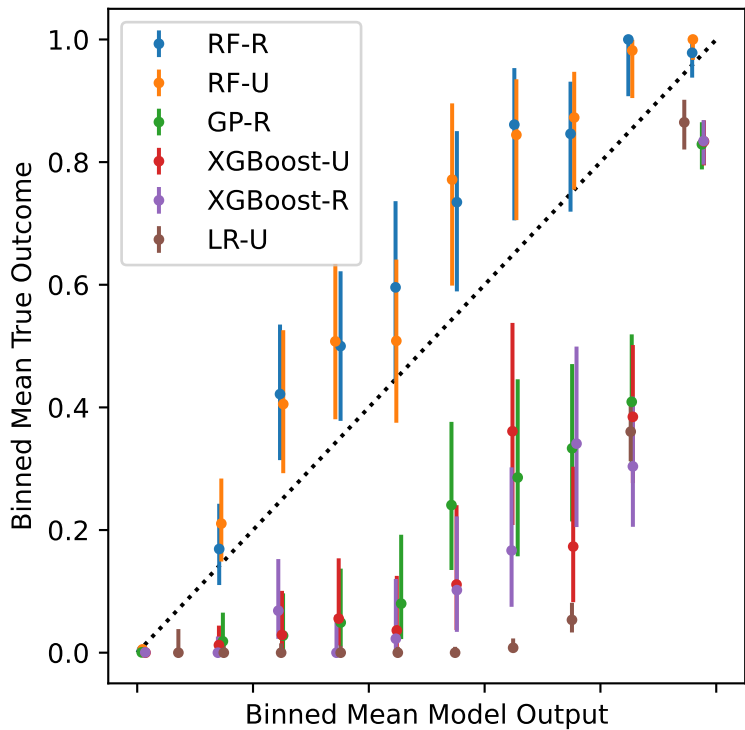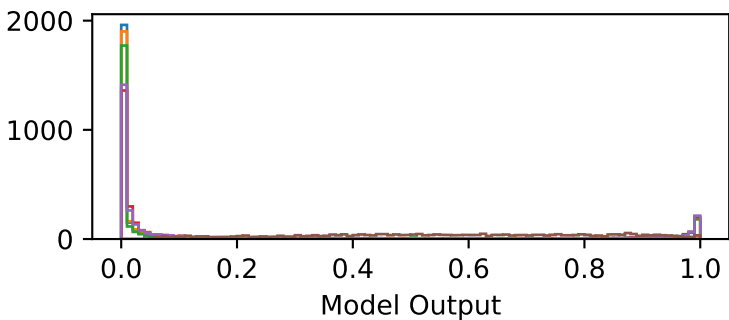

Supplement: Supplementary file 2 — Supplementary file2 (PDF 21 KB) [file 12028_2024_2119_MOESM2_ESM.pdf]

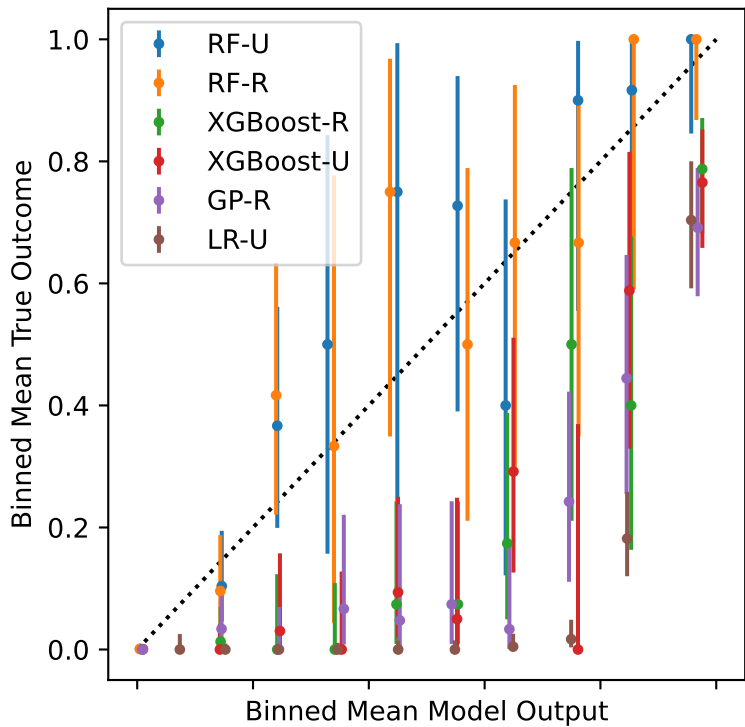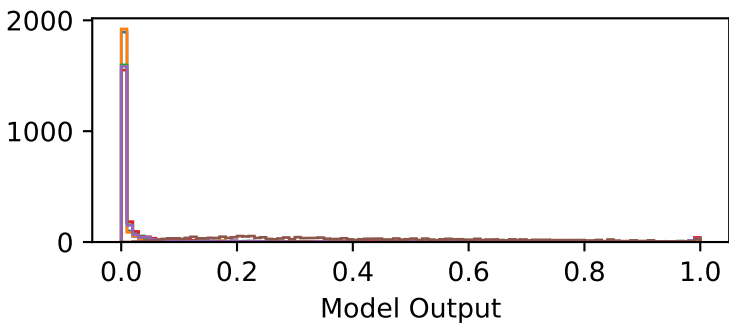

Supplement: Supplementary file 3 — Supplementary file3 (PDF 20 KB) [file 12028_2024_2119_MOESM3_ESM.pdf]

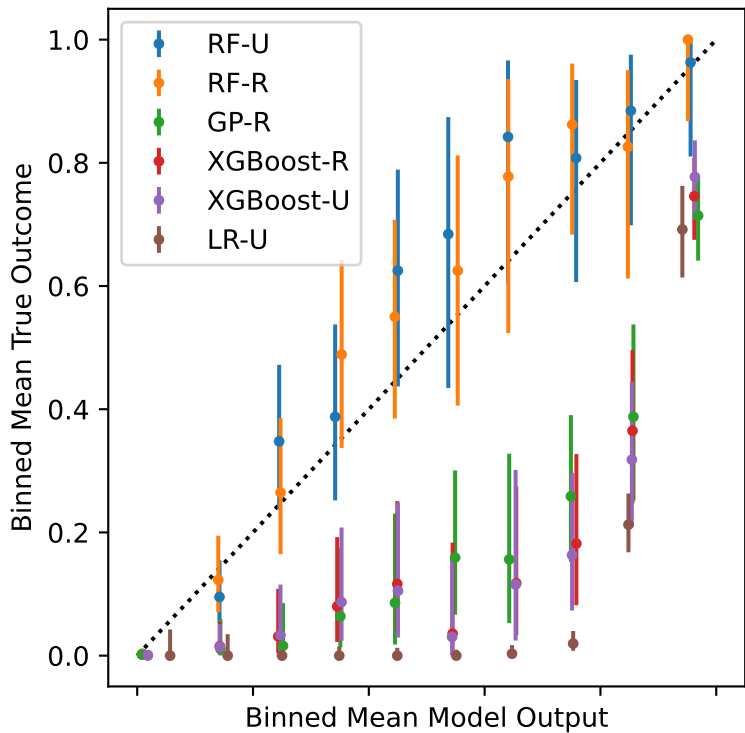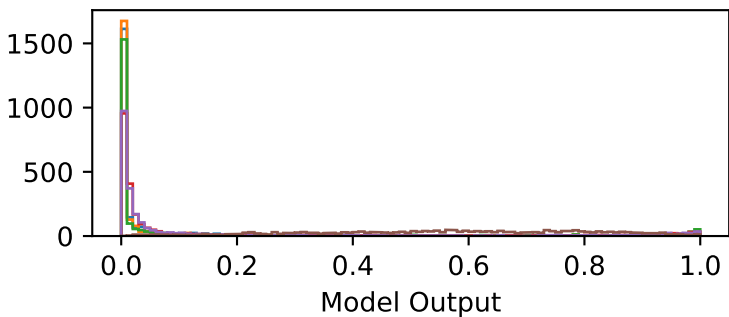

Supplement: Supplementary file 4 — Supplementary file4 (PDF 21 KB) [file 12028_2024_2119_MOESM4_ESM.pdf]

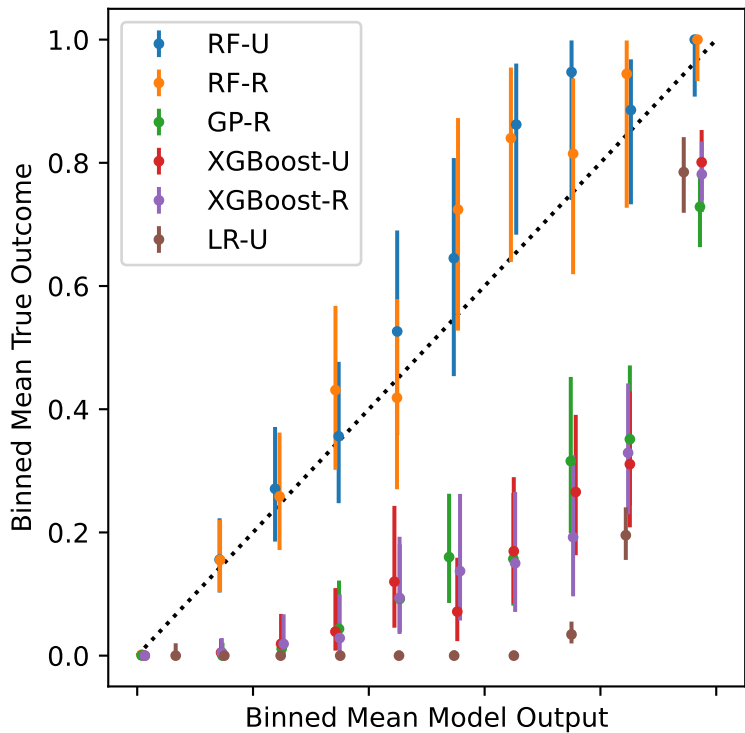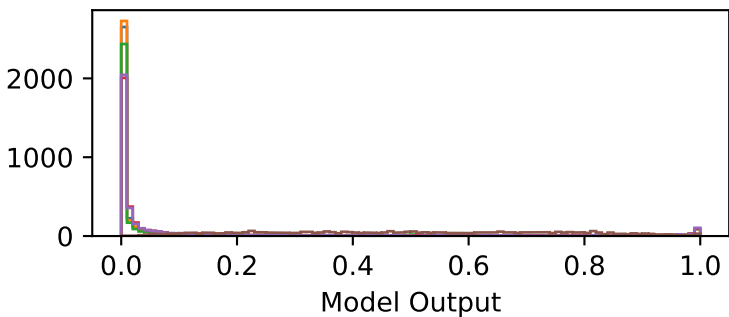

Supplement: Supplementary file 5 — Supplementary file5 (PDF 21 KB) [file 12028_2024_2119_MOESM5_ESM.pdf]

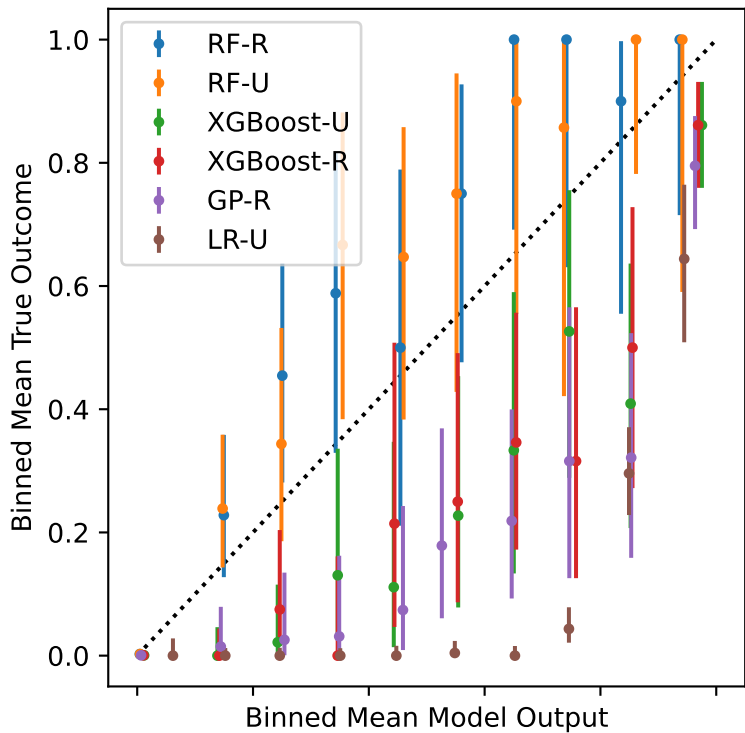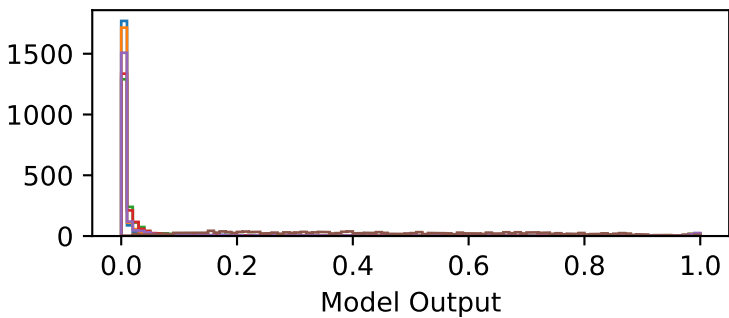

Supplement: Supplementary file 6 — Supplementary file6 (PDF 21 KB) [file 12028_2024_2119_MOESM6_ESM.pdf]

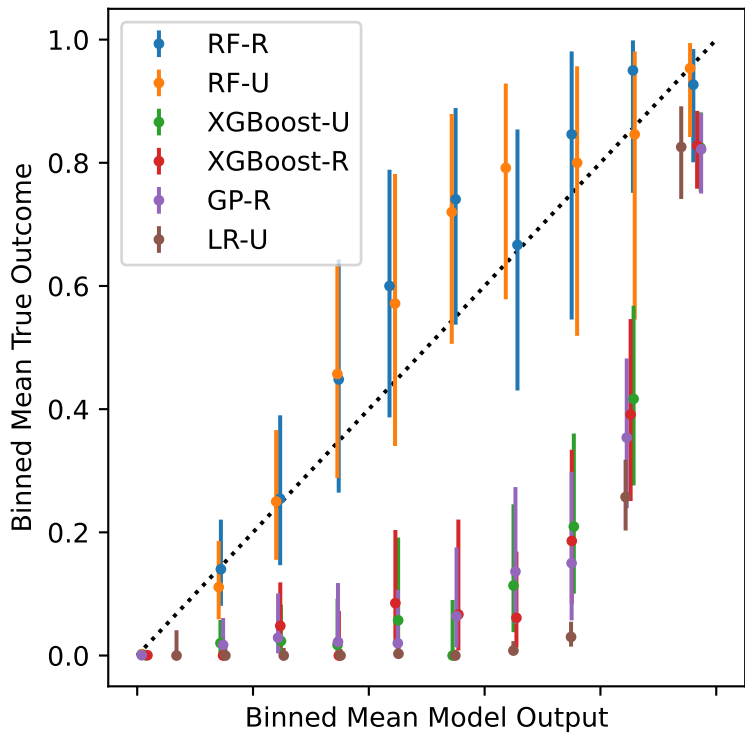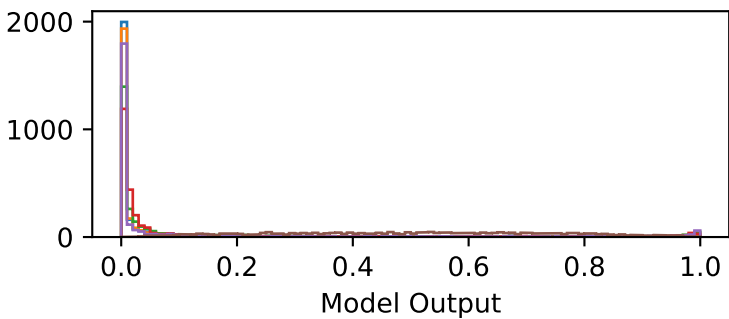

Supplement: Supplementary file 7 — Supplementary file7 (PDF 21 KB) [file 12028_2024_2119_MOESM7_ESM.pdf]

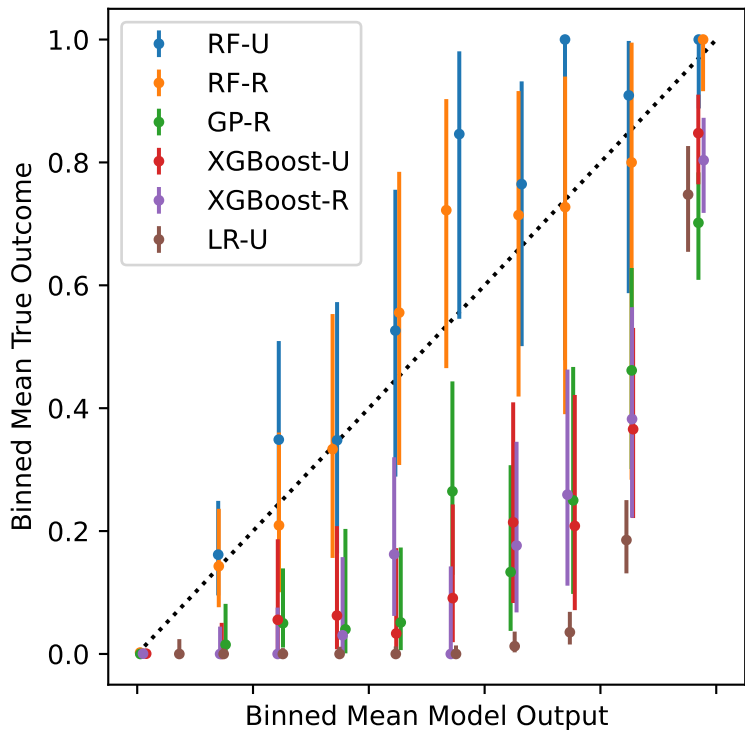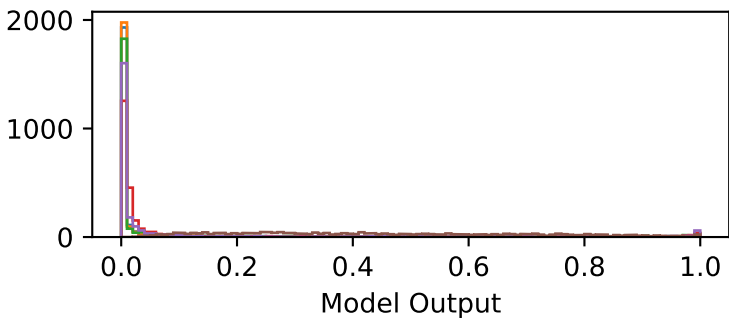

Supplement: Supplementary file 8 — Supplementary file8 (PDF 21 KB) [file 12028_2024_2119_MOESM8_ESM.pdf]

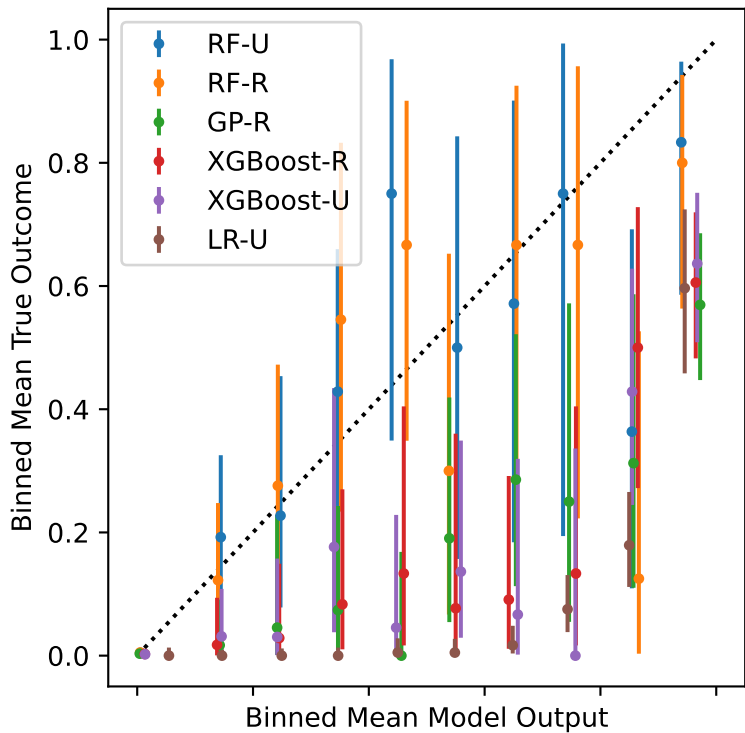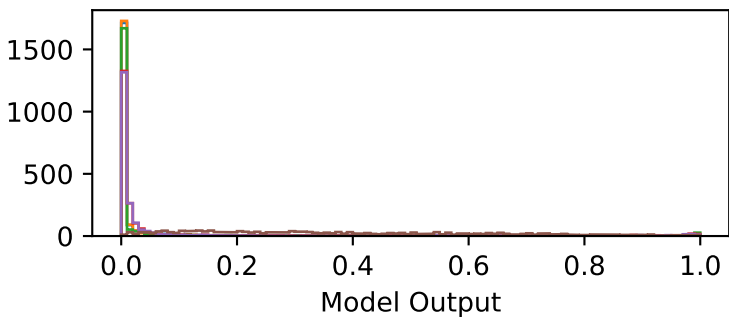

Supplement: Supplementary file 9 — Supplementary file9 (PDF 21 KB) [file 12028_2024_2119_MOESM9_ESM.pdf]

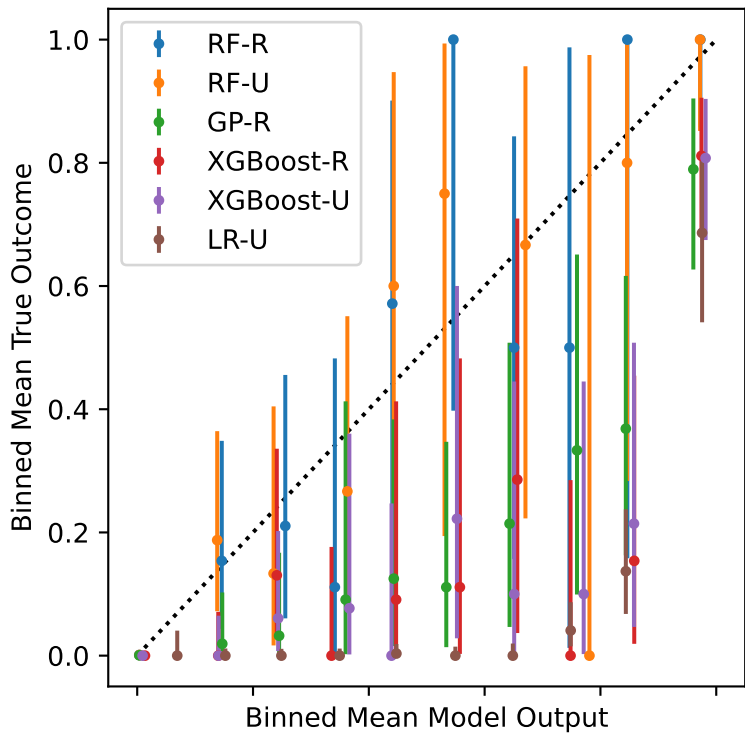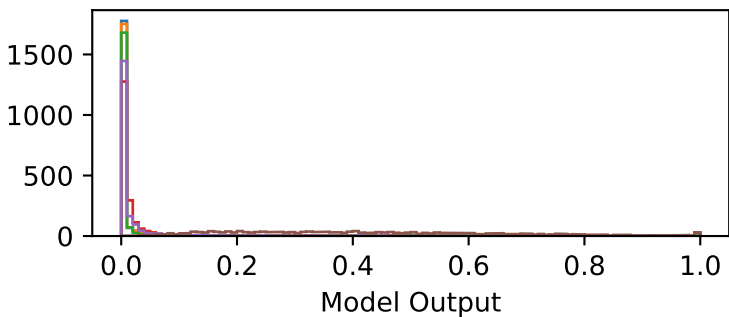

Supplement: Supplementary file 10 — Supplementary file10 (PDF 20 KB) [file 12028_2024_2119_MOESM10_ESM.pdf]

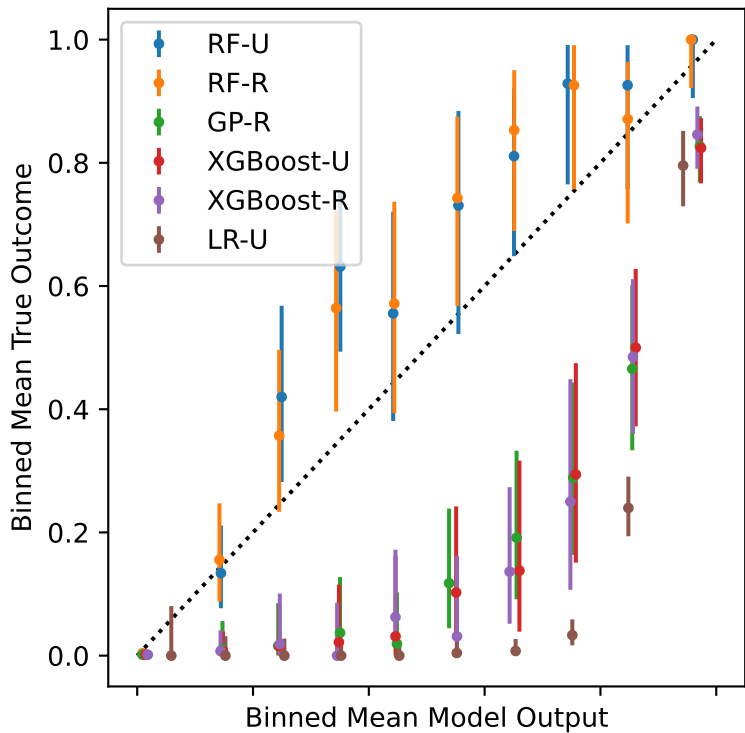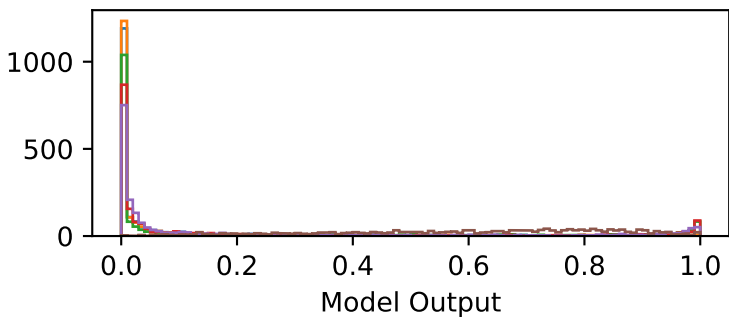

Supplement: Supplementary file 11 — Supplementary file11 (PDF 22 KB) [file 12028_2024_2119_MOESM11_ESM.pdf]

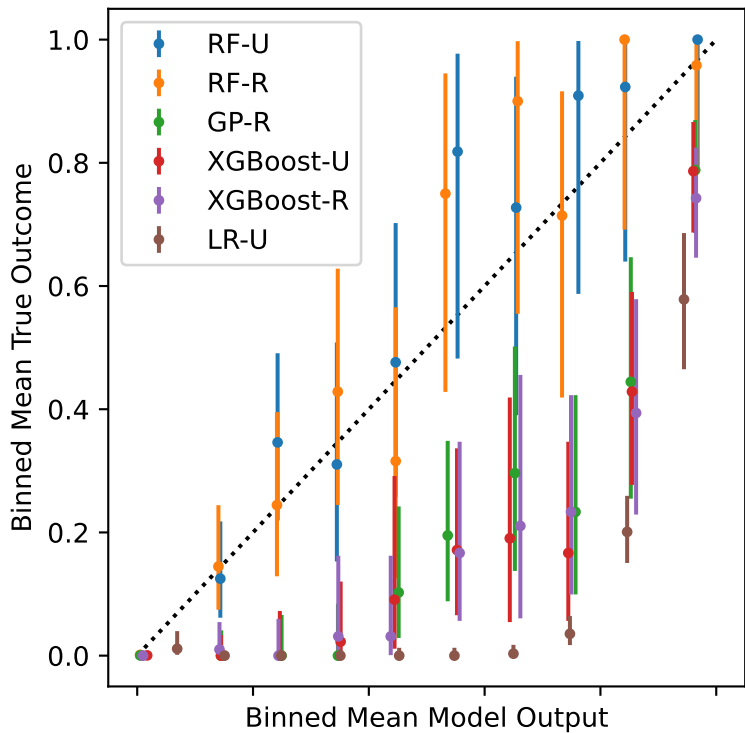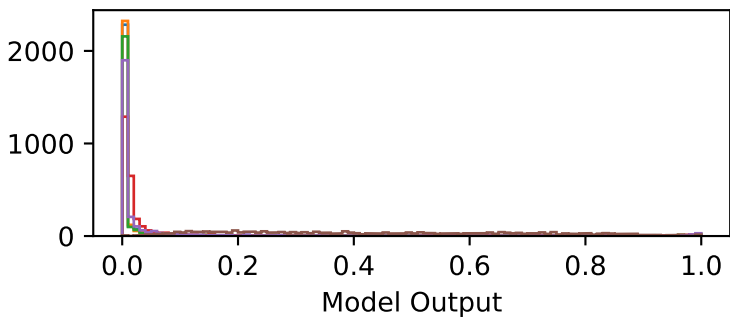

Supplement: Supplementary file 12 — Supplementary file12 (PDF 20 KB) [file 12028_2024_2119_MOESM12_ESM.pdf]

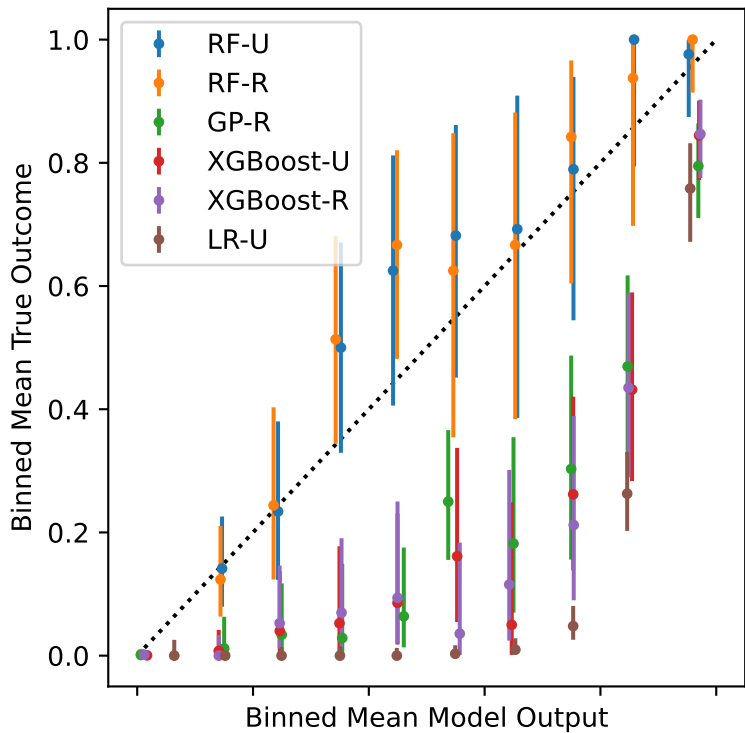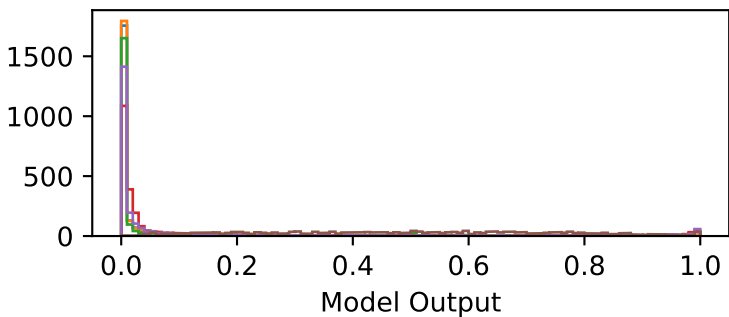

Supplement: Supplementary file 13 — Supplementary file13 (PDF 21 KB) [file 12028_2024_2119_MOESM13_ESM.pdf]

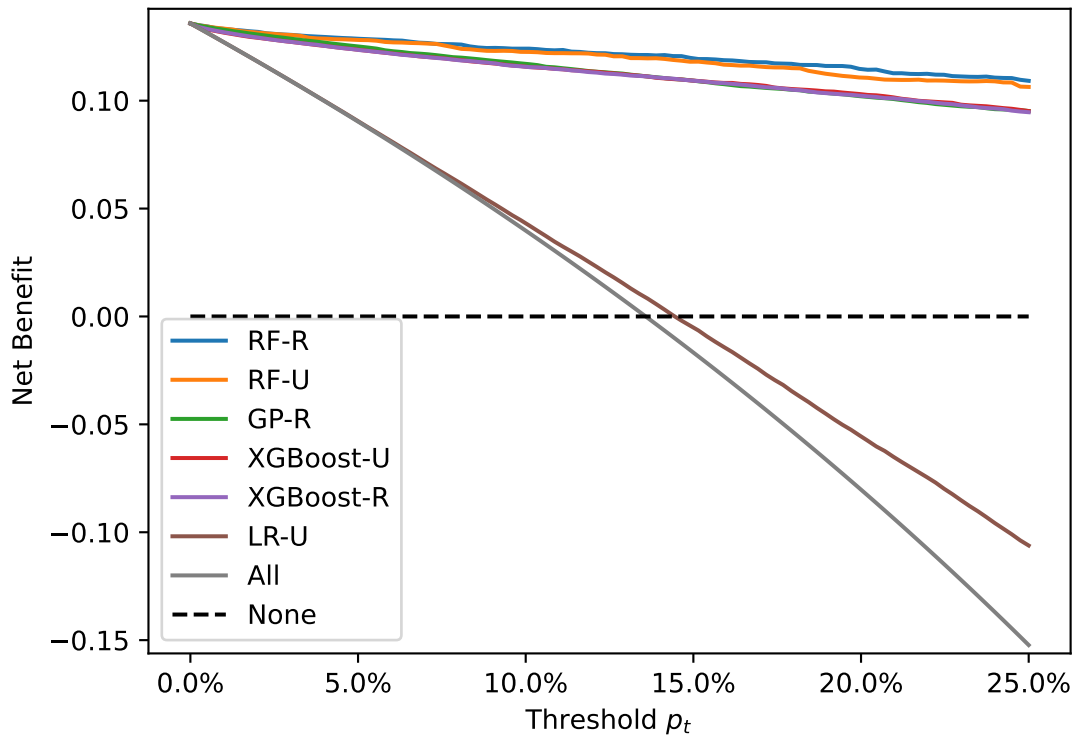

Supplement: Supplementary file 14 — Supplementary file14 (PDF 20 KB) [file 12028_2024_2119_MOESM14_ESM.pdf]

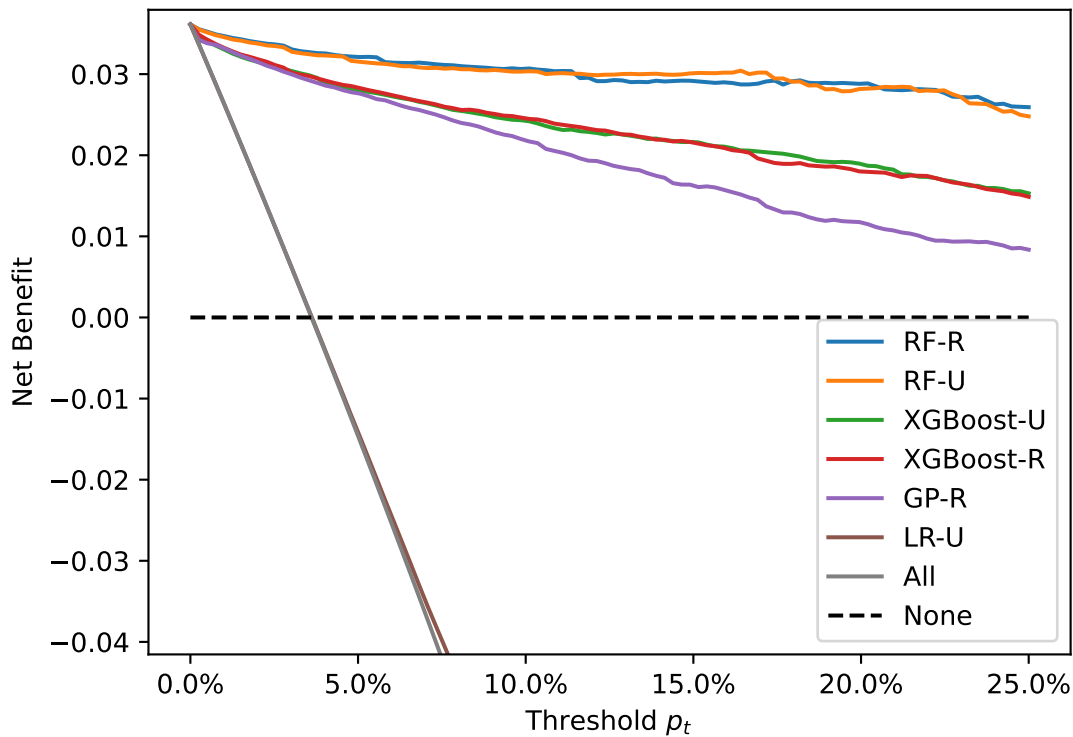

Supplement: Supplementary file 15 — Supplementary file15 (PDF 20 KB) [file 12028_2024_2119_MOESM15_ESM.pdf]

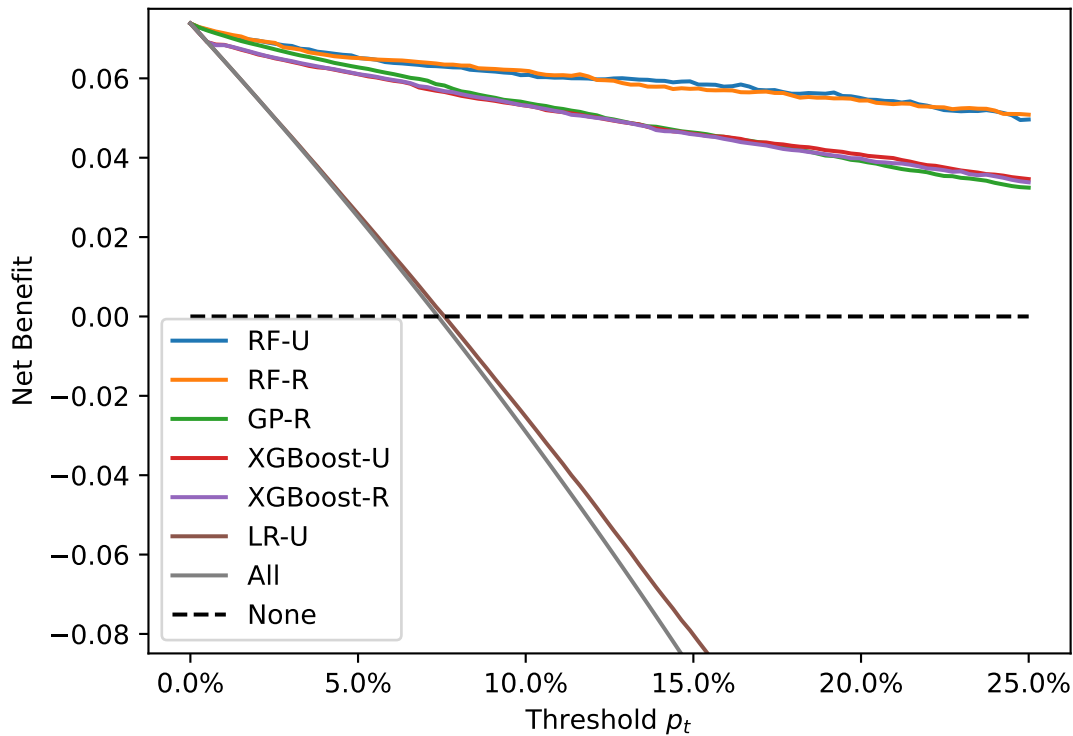

Supplement: Supplementary file 16 — Supplementary file16 (PDF 21 KB) [file 12028_2024_2119_MOESM16_ESM.pdf]

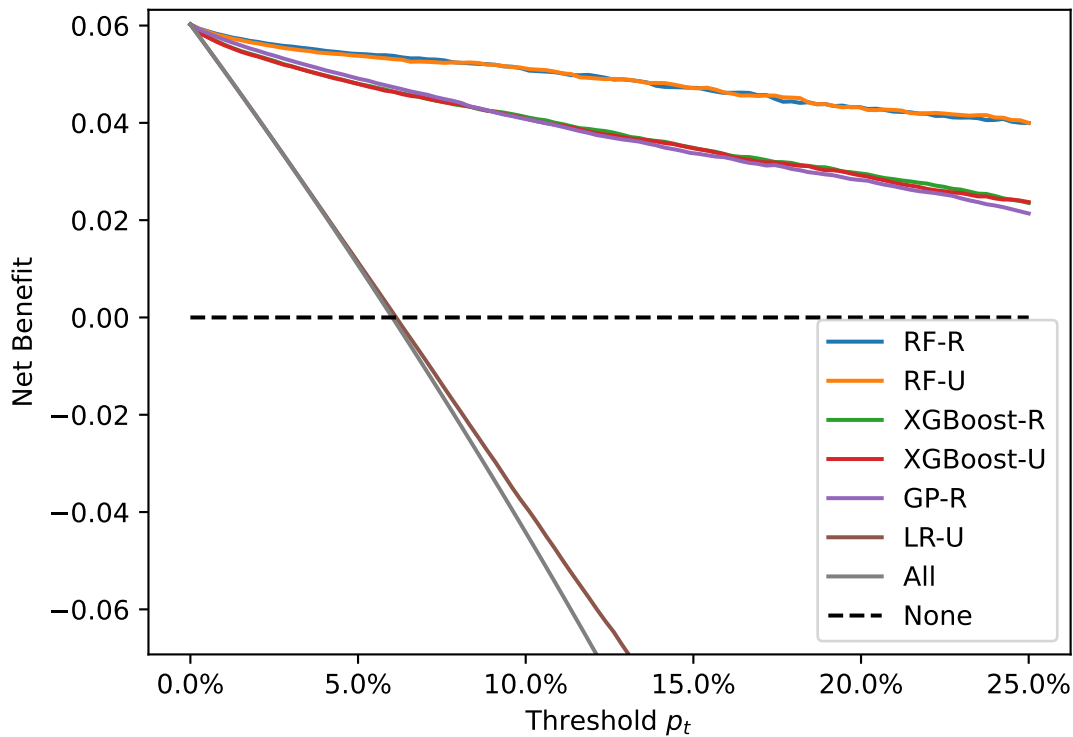

Supplement: Supplementary file 17 — Supplementary file17 (PDF 21 KB) [file 12028_2024_2119_MOESM17_ESM.pdf]

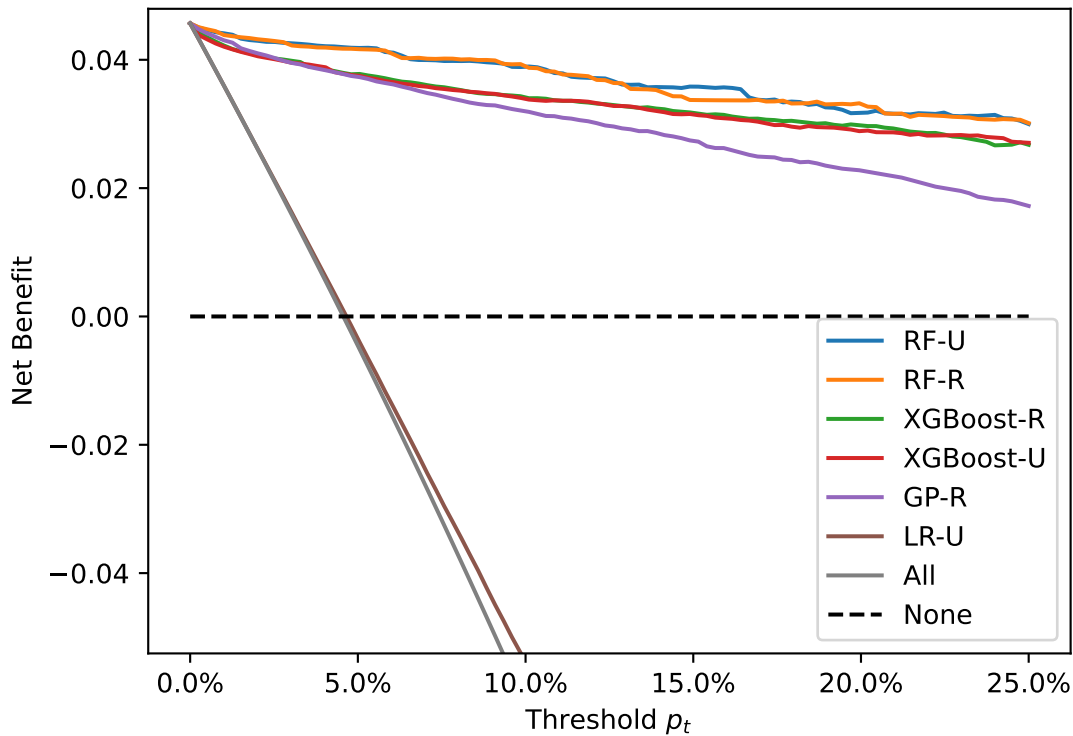

Supplement: Supplementary file 18 — Supplementary file18 (PDF 20 KB) [file 12028_2024_2119_MOESM18_ESM.pdf]

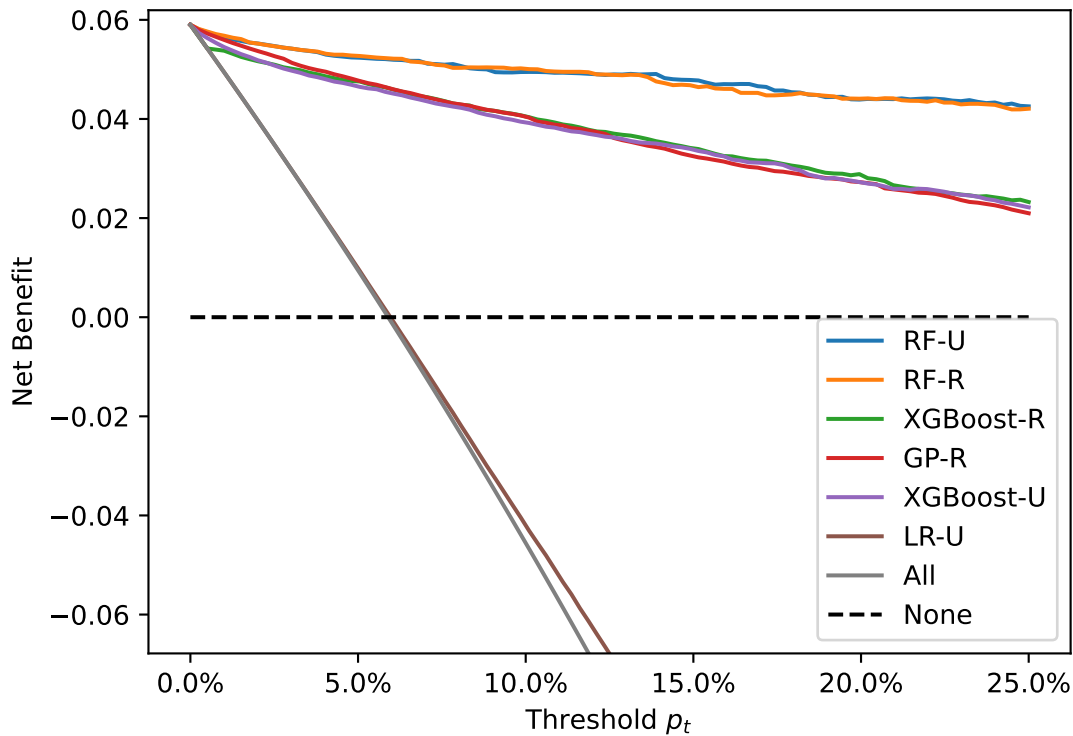

Supplement: Supplementary file 19 — Supplementary file19 (PDF 21 KB) [file 12028_2024_2119_MOESM19_ESM.pdf]

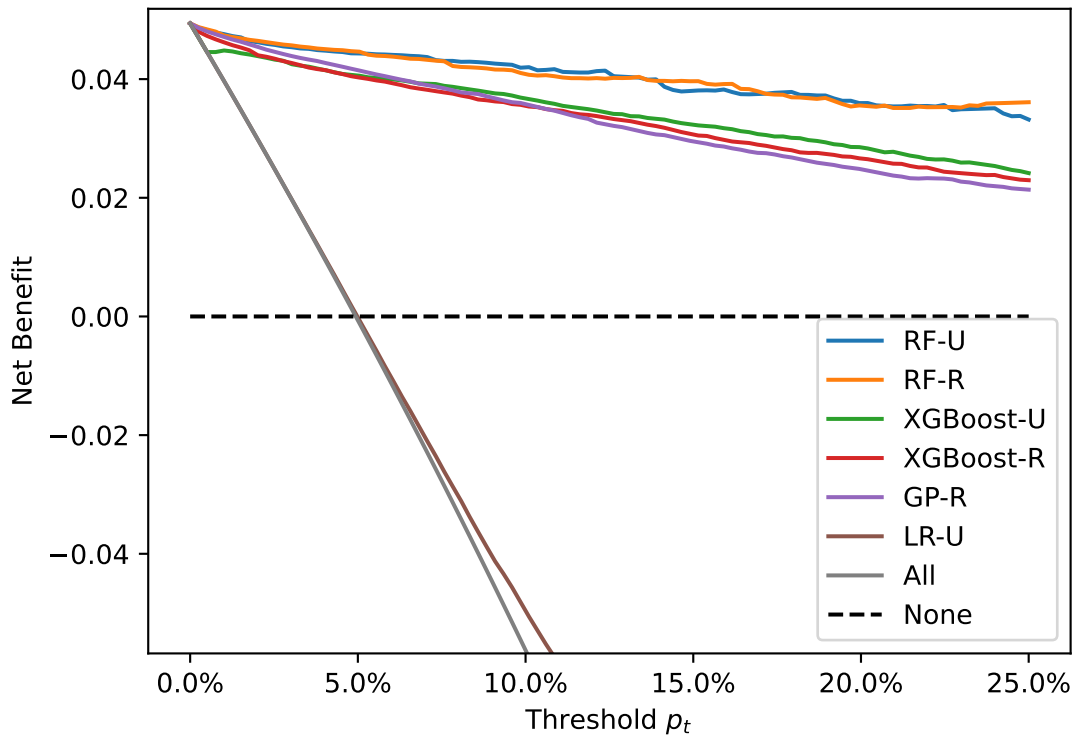

Supplement: Supplementary file 20 — Supplementary file20 (PDF 20 KB) [file 12028_2024_2119_MOESM20_ESM.pdf]

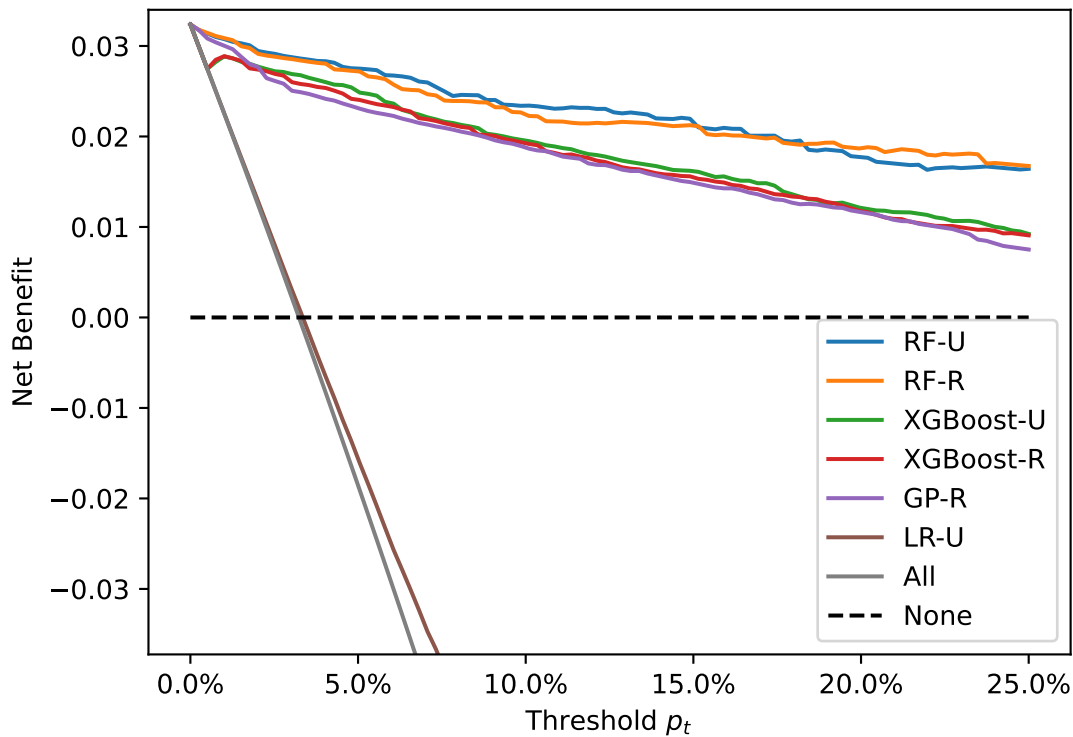

Supplement: Supplementary file 21 — Supplementary file21 (PDF 20 KB) [file 12028_2024_2119_MOESM21_ESM.pdf]

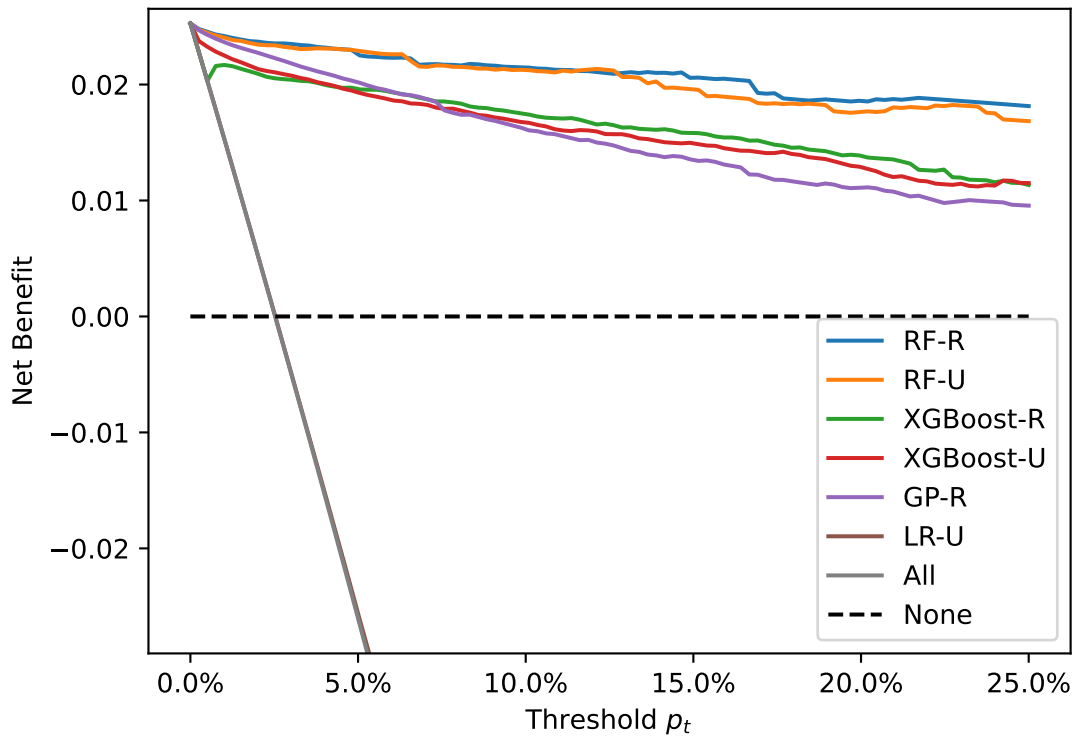

Supplement: Supplementary file 22 — Supplementary file22 (PDF 19 KB) [file 12028_2024_2119_MOESM22_ESM.pdf]

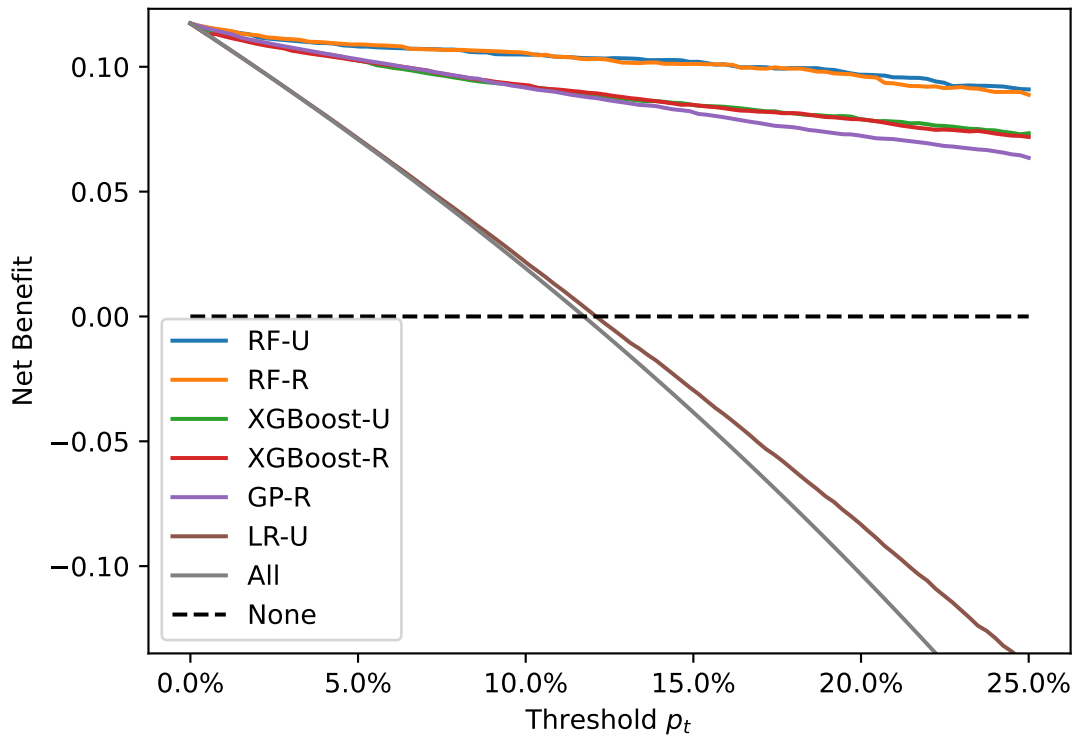

Supplement: Supplementary file 23 — Supplementary file23 (PDF 20 KB) [file 12028_2024_2119_MOESM23_ESM.pdf]

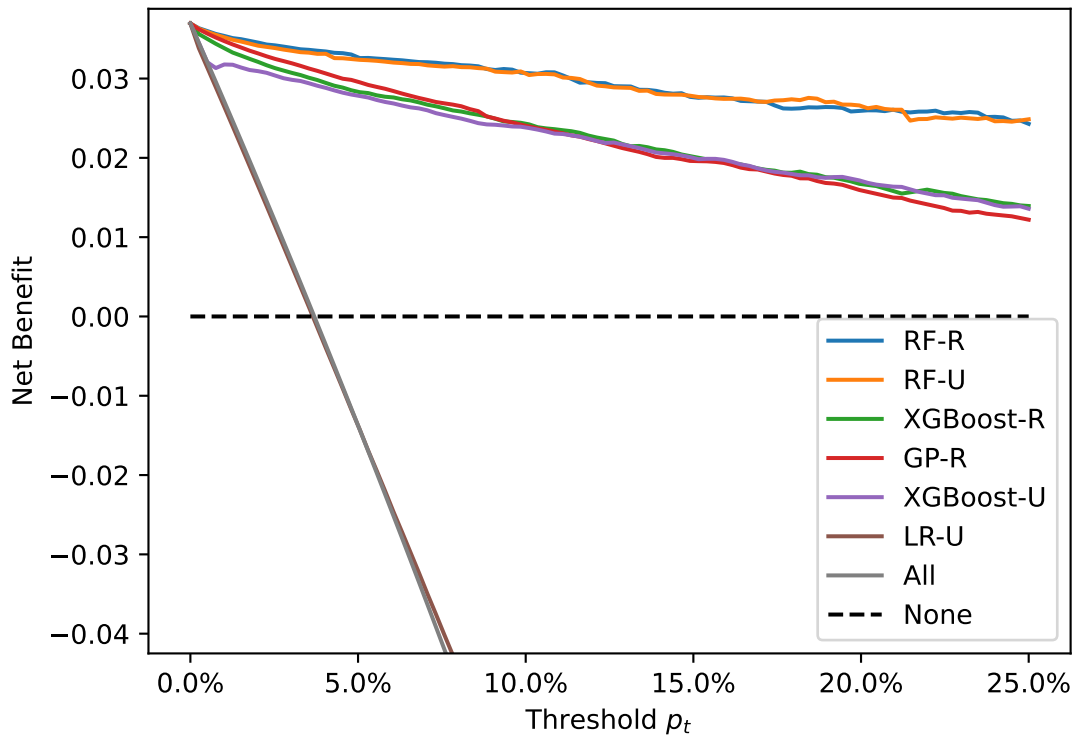

Supplement: Supplementary file 24 — Supplementary file24 (PDF 20 KB) [file 12028_2024_2119_MOESM24_ESM.pdf]

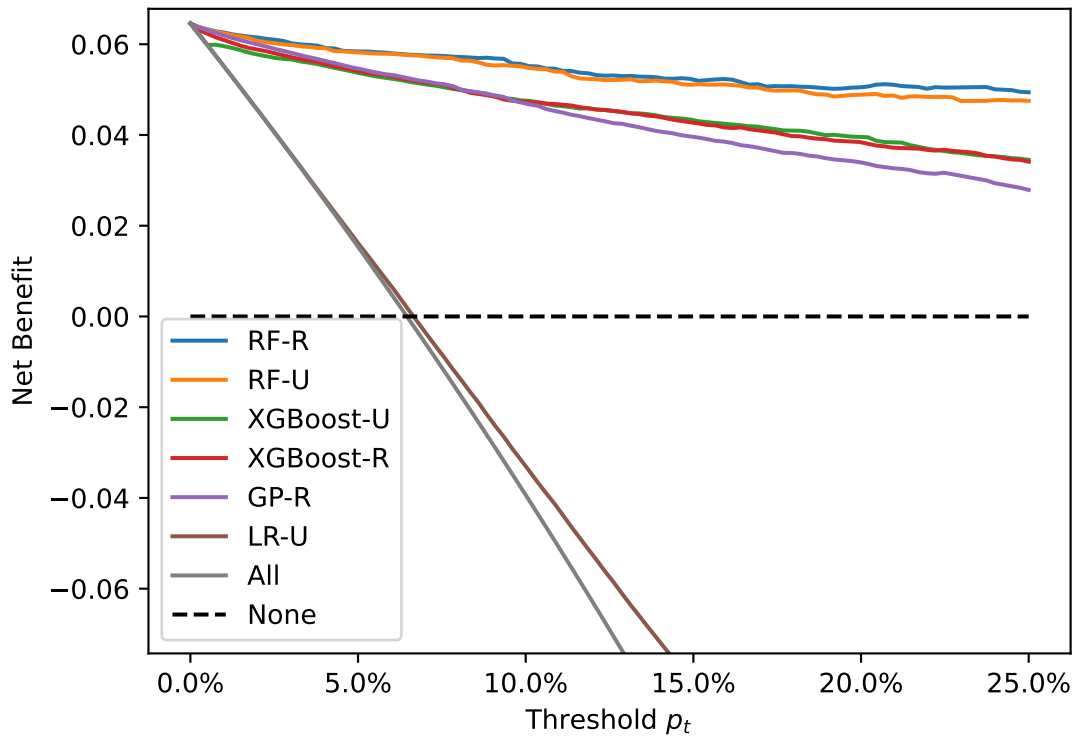

Supplement: Supplementary file 25 — Supplementary file25 (PDF 21 KB) [file 12028_2024_2119_MOESM25_ESM.pdf]

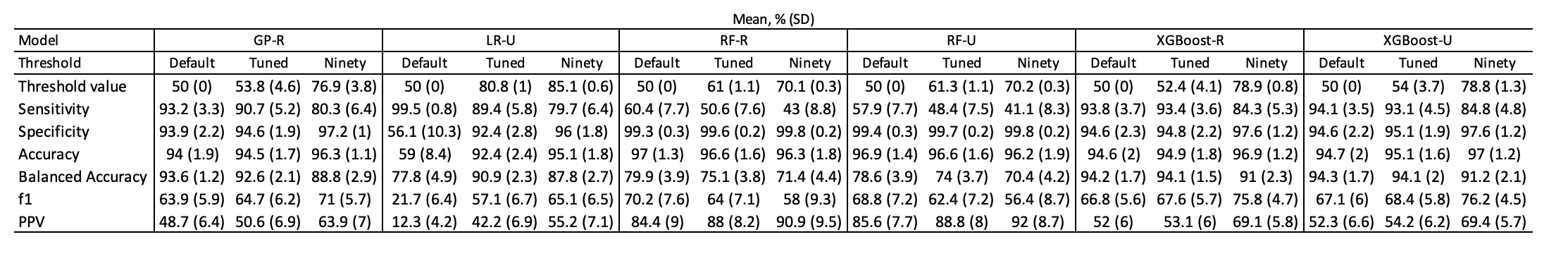

Supplement: Supplementary file 26 — Supplementary file25 (TIFF 3309 KB) [file 12028_2024_2119_MOESM26_ESM.tiff]

# Equator checklist

#
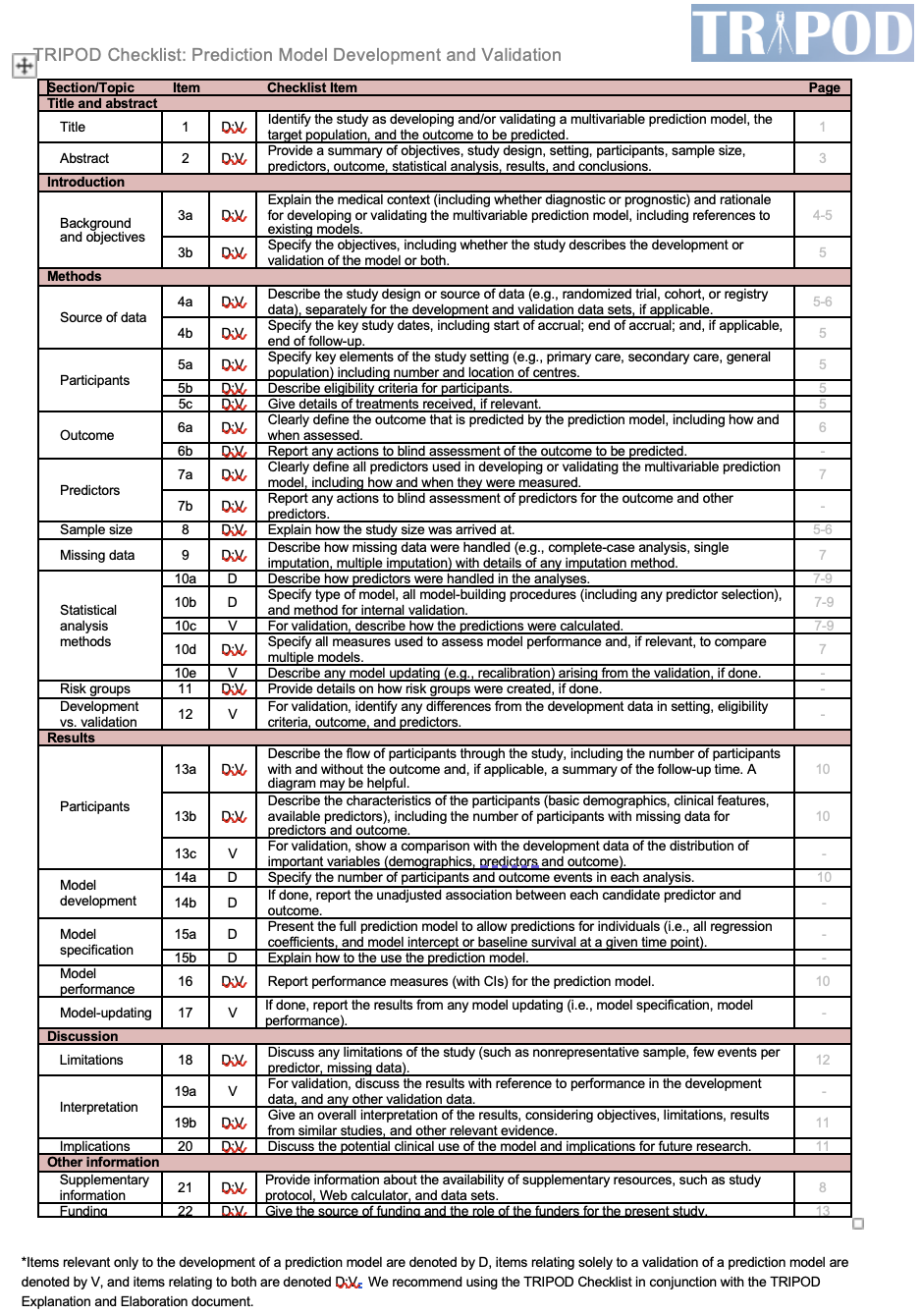

Supplement: Supplementary file 28 — Supplementary file28 (DOCX 455 KB) [file 12028_2024_2119_MOESM28_ESM.docx]
