# Supplementary material for: Machine Learning Based Prediction of Imminent ICP Insults During Neurocritical Care of Traumatic Brain Injury
Source: Neurocrit Care. 2024 Sep 25;42(2):387–97. doi: 10.1007/s12028-024-02119-7 (PMC11950052; doi:10.1007/s12028-024-02119-7)
Supplement: Supplementary file 27 — Supplementary file27 (DOCX 28 KB) [file 12028_2024_2119_MOESM27_ESM.docx]

# Appendix – Features and Data

| **Number of features** | **Description** | **Feature set** | **Comment** |
| --- | --- | --- | --- |
| 3*240 = 720 | Minute-by-minute values for ICPm, ABPm and CPPm | Restricted^1^ |  |
| 2*12*3 = 72 | Mean + SD over 5 min windows last 1 hour for ICPm, ABPm and CPPm | Restricted^1^ |  |
| 2*12*3 = 72 | Mean + SD over 10 min windows last 2 hour for ICPm, ABPm and CPPm | Restricted^1^ |  |
| 2*12*3 = 72 | Mean + SD over 20 min windows last 4 hour for ICPm, ABPm and CPPm | Restricted^1^ |  |
| 1*3 =3 | Difference between the first and last value within the 4h window for ICPm, ABPm and CPPm | Restricted^1^ |  |
| 5*3 = 15 | Five first cepstrum coefficients of the signal for the last 4 hours for ICPm, ABPm and CPPm | Restricted^1^ |  |
| 5*3 = 15 | Value of the 5 largest fourier coefficients of the signal for the last 4 hours for ICPm, ABPm and CPPm | Restricted^1^ |  |
| 5*3 = 15 | Frequency of the 5 largest fourier coefficients of the signal for the last 4 hours for ICPm, ABPm and CPPm | Restricted^1^ |  |
| 24 | ICPm-ABPm correlation coefficients computed over 10-minute windows for the last 4 hours | Restricted^1^ |  |
| 23 | 1st difference of the ICPm-ABPm correlation signal | Restricted^1^ |  |
| 5*2 | First 5 cepstrum coefficients of the ICPm-ABPm correlation signal. | Restricted^1^ | Included twice for consistency with the electronic supplement^1^ |
| 2*2*240 = 960 | Minute-by-minute values for ICPd, ICPs, ABPd, ABPs | Unrestricted | Approximates diastolic and systolic pressures |
| 240 | Minute-average Heart Rate (HR). Based on current HR calculated from the last 8 heart beats | Unrestricted | Beat detection via Pan Tomkins algorithm ^2^ and ECG data |
| 240 | Heart Rate Variability (HRV)  5-minute SDRR (SD of r-r intervals). 5 minute moving window. | Unrestricted | Outlier removal is employed, removing inter beat intervals that are too large, similar to the description in ^3^. While they compute medians over a 2sided 25-long window, we only apply a left sided window to allow online filtering with no time delay. |
| 240 | Respiratory Rate (RR)  Minute mean respiratory rate | Unrestricted | Estimated from ECG by:   - Locating the Q and R peaks for each heart beat - Compute the beat amplitude (ECG value at R minus value at Q) - Resample to 4 Hz by linear interpolation - Compute short-time fourier transform over 5 minute windows, 30 seconds hop length, hamming window, - Find domimant frequency between 5 and 30 bpm   The method is inspired by the amplitude methods of ^4^. |
| 240 | 1 if any data was missing a certain minute, otherwise 0, for the last 4 hours. | Unrestricted and Restricted |  |
| 1 | Length of monitoring | Unrestricted | How many decimal hours since start of monitoring data. |
| 1 | RLS-85  1/2/3a/3b/4/5/6/7/8 | Unrestricted | Ordinal encoded 1-9 |
| 2*1 | Pupil Left + Right   - Normal - Sluggish - Fixed | Unrestricted | Ordinal encoded 1-3 |
| 1 | Motor Response   - No motor response - Extension to pain - Flexion to pain - Withdrawal from pain - Localizing pain - Obeys Commands | Unrestricted | Ordinal encoded 1-6 |
| 1 | Age | Unrestricted | Mean age of the age group that the patient was assigned to |
| 1 | Sex   - Male - Female | Unrestricted |  |
| 2 | Anticoagulants   - Yes - No - Unknown | Unrestricted | One hot encoded with reference category |
| 7 | CT Findings   - Acute SDH - Contusions - Mixed - Diffuse Axonal Injury (DAI) - Traumatic SAH - EDH - Other - Impression fracture | Unrestricted | One hot encoded with reference category |
| 2 | Verbal Response   - Not Applicable - No Verbal Response - Incomprehensible sounds / Inappropriate words / Confused / Oriented | Unrestricted | One hot encoded with reference category |
| 4 | Eye Response   - No eye opening - Eye opening to pain - Eye opening to verbal command - Eyes open spontaneously - Not Applicable | Unrestricted | One hot encoded with reference category. |

ABP = Arterial Blood Pressure. Raw data 100Hz

ICP = Intracranial Pressure. Raw data 100 Hz

ECG = Electrocardiogram. Raw data 200 Hz

XXXd = minute-minimum value of XXX

XXXs = minute-maximum value of XXX

XXXm = minute-mean value of XXX

CPP = Cerebral Perfusion Pressure. CPPm = ICPm – ABPm.

In all series, missing data is replaced by 0.

# References

1. Güiza F, Depreitere B, Piper I, Van den Berghe G, Meyfroidt G. Novel Methods to Predict Increased Intracranial Pressure During Intensive Care and Long-Term Neurologic Outcome After Traumatic Brain Injury: Development and Validation in a Multicenter Dataset. Crit Care Med 2013;41(2):554–64.

2. Pan J, Tompkins WJ. A Real-Time QRS Detection Algorithm. IEEE Trans Biomed Eng 1985;BME-32(3):230–6.

3. Cajal D, Hernando D, Lázaro J, Laguna P, Gil E, Bailón R. Effects of Missing Data on Heart Rate Variability Metrics. Sensors 2022;22(15):5774.

4. Ruangsuwana R, Velikic G, Bocko M. Methods to extract respiration information from ECG signals [Internet]. In: 2010 IEEE International Conference on Acoustics, Speech and Signal Processing. Dallas, TX, USA: IEEE; 2010 [cited 2023 Nov 2]. p. 570–3.Available from: http://ieeexplore.ieee.org/document/5495584/
